# Supplementary material for: Controlled magnetic bistability of a helical non-Kekulé hydrocarbon on a Au(111) surface
Source: Nat Commun. 2026 Jan 9;17:331. doi: 10.1038/s41467-025-67039-2 (PMC12789080; doi:10.1038/s41467-025-67039-2)
Supplement: Supplementary file 1 — Supplementary Information [file 41467_2025_67039_MOESM1_ESM.pdf]

Supplementary information for

Controlled magnetic bistability of a helical non-Kekulé hydrocarbon on a Au(111) surface

Table of contents

1 Synthesis 2

2 Nuclear magnetic resonance (NMR) spectroscopy 10

3 Mass spectrometry 21

4 X-ray crystallography 27

5 Additional STM data 30

6 Additional computational data 35

7 References 36

Open access data

The raw NMR and IR data are available free of charge on a public repository Zenodo under the link https://zenodo.org/record/8253265 (DOI: 10.5281/zenodo.8253265).

1 Synthesis

General information.

Glassware utilized in the reactions, carried out under both anhydrous and non-anhydrous conditions, was cleaned and dried in an oven at 150 °C for at least 24 hours prior to the experiment. All reagents and solvents, including non-anhydrous and anhydrous solvents such as CH_2Cl_2, cyclohexane, EtOAc or THF, were supplied from commercial sources and used without additional purification unless otherwise noted. Thin-layer chromatography (TLC) was used to monitor the reactions, using aluminium sheets covered with silica gel containing fluorescent indicator UV_{254} (available from Alugram SIL G/UV_{254}, Macherey-Nagel or Sigma-Aldrich) and viewed under UV light (254 or 365 nm). Silica gel 60 (230–400 mesh, Sigma-Aldrich) was used for flash column chromatography.

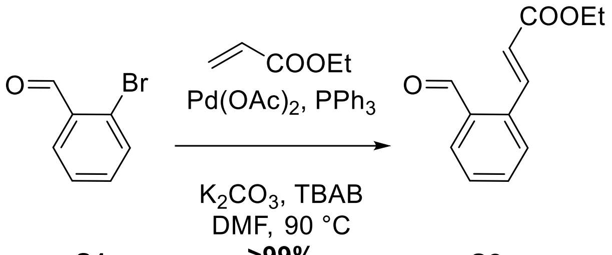

Ethyl (*E*)-3-(2-formylphenyl)acrylate (S2).

The reaction was carried out under inert conditions according to a modified literature procedure[1]. In a pre-dried argon-flushed Schlenk tube, a mixture of 2-bromobenzaldehyde (**S1**; 1.59 g, 8.59 mmol), $Pd(OAc)_2$ (579 mg, 2.58 mmol), PPh_3 (1.35 g, 5.15 mmol), K_2CO_3 (2.37 g, 17.2 mmol) and tetrabutylammonium bromide (TBAB; 5.54 g, 77.3 mmol) in DMF (50 mL) was deoxygenated by freeze-pump-thaw technique in three cycles. Ethyl acrylate (8.41 mL, 77.3 mmol, deoxygenated by sonication under argon atmosphere) was added and the mixture was heated at 90 °C for 20 h. Then, CH_2Cl_2 (100 mL) was added and the organic phase was washed with water and aqueous NH_4Cl (sat.), dried over anhydrous $MgSO_4$ and concentrated under reduced pressure. The crude product was purified by flash column

chromatography (SiO_2, cyclohexane/EtOAc 9:1, v/v) to yield the desired product as a colourless oil (1.75 g, >99%).

^1H NMR (400 MHz, $CDCl_3$, ppm): δ 10.31 (s, 1H), 8.52 (d, J = 15.9 Hz, 1H), 7.89 (d, J = 7.5 Hz, 1H), 7.66–7.53 (m, 3H), 6.38 (d, J = 16.0 Hz, 1H), 4.30 (q, J = 7.1 Hz, 2H), 1.35 (t, J = 7.1 Hz, 3H). The data is in agreement with those from literature[2].

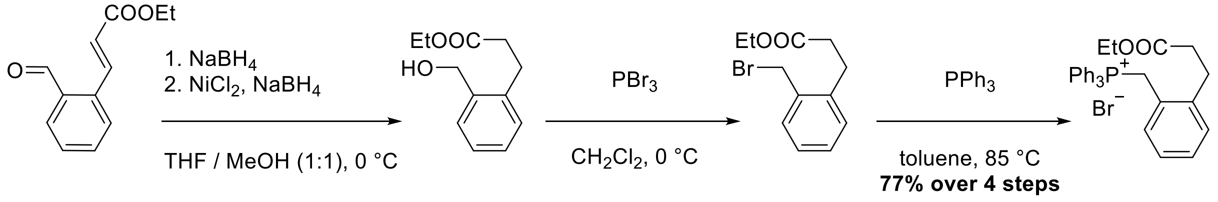

(2-(3-Ethoxy-3-oxopropyl)benzyl)triphenylphosphonium bromide (4).

To a solution of ethyl (*E*)-3-(2-formylphenyl)acrylate (**S2**; 3.62 g, 17.7 mmol) in THF/MeOH (1.2 L, 1:1), $NaBH_4$ (670 mg, 17.7 mmol) was added at 0 °C and the mixture was stirred at the same temperature for 10 min. Then, $NiCl_2 \cdot 6H_2O$ (9.26 g, 38.9 mmol) and $NaBH_4$ (2.68 g, 70.8 mmol) were added in small portions at 0 °C and the mixture was stirred at the same temperature for additional 15 min before it was concentrated under reduced pressure. The crude product was dissolved in CH_2Cl_2 (500 mL), filtered through a pad of silica and concentrated under reduced pressure to afford the desired crude intermediate **S3** as a colourless oil (3.69 g).

The next step was carried out under inert conditions. To a solution of the alcohol intermediate **S3** (3.69 g, 17.7 mmol) in CH_2Cl_2 (120 mL), PBr_3 (66.5 µL, 7.08 mmol) was added dropwise at 0 °C and the mixture was allowed to warm to room temperature over 30 min before aqueous $NaHCO_3$ (10 mL, sat.) was added. The aqueous phase was extracted with CH_2Cl_2 (2 × 10 mL) and the combined organic layers were dried over anhydrous $MgSO_4$, filtered and concentrated under reduced pressure to yield the desired crude intermediate **S4** (3.70 g).

To a solution of the bromide intermediate **S4** (3.70 g, 13.6 mmol) in toluene (100 mL), PPh_3 (3.92 g, 15.0 mmol) was added at room temperature and the mixture was stirred at 85 °C for

24 h. The resulting suspension was cooled down to room temperature, filtered and washed with toluene and then Et_2O to afford the desired product **4** as a white solid (5.61 g, 77%).

^{1}H NMR (400 MHz, $CDCl_3$, ppm): δ 7.82–7.75 (m, 3H), 7.72–7.60 (m, 12H), 7.30–7.26 (m, 1H), 7.24–7.18 (m, 1H), 7.04 (dd, J = 7.6, 7.6 Hz, 1H), 7.00 (d, J = 7.7 Hz, 1H), 5.41 (d, J = 14.2 Hz, 2H), 4.08 (q, J = 7.1 Hz, 2H), 2.35 (t, J = 7.6 Hz, 2H), 2.10 (t, J = 7.6 Hz, 2H), 1.21 (t, J = 7.1 Hz, 3H).

^{13}C NMR (101 MHz, $CDCl_3$, ppm): δ 172.9, 141.1 (d, J = 5.6 Hz), 135.2 (d, J = 3.1 Hz), 134.5 (d, J = 9.6 Hz), 132.4 (d, J = 4.9 Hz), 130.4 (d, J = 12.4 Hz), 129.6 (d, J = 3.3 Hz), 129.3 (d, J = 4.2 Hz), 127.6 (d, J = 3.5 Hz), 125.5, 117.9 (d, J = 85.4 Hz), 60.9, 34.9, 27.8 (d, J = 46.3 Hz), 27.0, 14.3.

^{31}P NMR (161 MHz, $CDCl_3$, ppm): δ 22.73.

HRMS (ESI) m/z: $[M]^+$ Calcd for $C_{30}H_{30}PO_2$ 453.19779; Found 453.19723.

IR (cm^{-1}): 2987, 1711, 1435, 1109, 744, 688, 499.

m.p. (°C): 189.4–190.8.

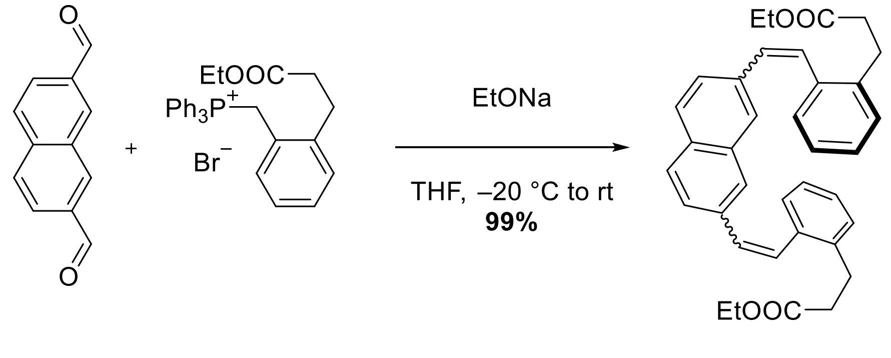

3 **4** **5**

Diethyl 3,3'-((naphthalene-2,7-diylbis(ethene-2,1-diyl))bis(2,1-phenylene))dipropionate (5).

The reaction was carried out under inert conditions. To a suspension of ethyl 3-(2(2-(3-ethoxy-3-oxopropyl)benzyl)triphenylphosphonium bromide (**4**; 3.67 g, 6.88 mmol) in THF (200 mL),

sodium ethoxide solution (2.51 mL, 6.73 mmol, 21% w/w in EtOH) was added dropwise at –20 °C and the mixture was stirred at the same temperature for 30 min. A solution of 2,7-naphthalenedicarbaldehyde[3] (**3**; 551 mg, 2.99 mmol) in THF (5 mL) was then added at –20 °C and the mixture was allowed to warm to 0 °C over 2 h, and further stirred at the same temperature for 1 h before it was poured into aqueous NH_4Cl (10 mL, sat.). The aqueous phase was extracted with CH_2Cl_2 (2 × 10 mL) and the combined organic layers were dried over anhydrous $MgSO_4$, filtered and concentrated under reduced pressure. The crude product was purified by flash column chromatography (SiO_2, cyclohexane/EtOAc 9:1, v/v) to afford the desired product **5** as a colourless oil (1.59 g, 99%) and as a mixture of three stereoisomers (*EE*, *ZZ*, *EZ*). For characterisation purposes, a portion of the product mixture was dissolved in toluene (100 mL) and, upon addition of I_2 (79.5 mg, 5% w/w), heated at reflux for 16 h before it was poured into aqueous $Na_2S_2O_3$ (30 mL, sat.). The aqueous phase was extracted with EtOAc (2 × 30 mL) and the combined organic layers were dried over anhydrous $MgSO_4$, filtered and concentrated under reduced pressure to give (*EE*)-**5** as a light-yellow solid.

^1H NMR (400 MHz, CD_2Cl_2, ppm): δ 7.91 (d, J = 1.7 Hz, 2H), 7.85 (d, J = 8.6 Hz, 2H), 7.78 (dd, J = 8.5, 1.7 Hz, 2H), 7.71 (dd, J = 6.7, 1.5 Hz, 2H), 7.56 (d, J = 16.0 Hz, 2H), 7.33–7.24 (m, 6H), 7.23 (d, J = 16.3 Hz, 2H), 4.13 (q, J = 7.1 Hz, 4H), 3.17 (t, J = 7.9 Hz, 4H), 2.65 (t, J = 7.9 Hz, 4H), 1.24 (t, J = 7.2 Hz, 6H).

^{13}C NMR (101 MHz, CD_2Cl_2, ppm): δ 173.0, 139.0, 136.4, 136.0, 134.4, 133.1, 130.9, 130.0, 128.5, 128.2, 127.3, 127.2, 126.6, 126.2, 124.1, 60.8, 36.3, 28.9, 14.4.

HRMS (ESI) *m*/*z*: $[M + H]^+$ Calcd for $C_{36}H_{36}O_4$ 533.26864; Found 533.26902.

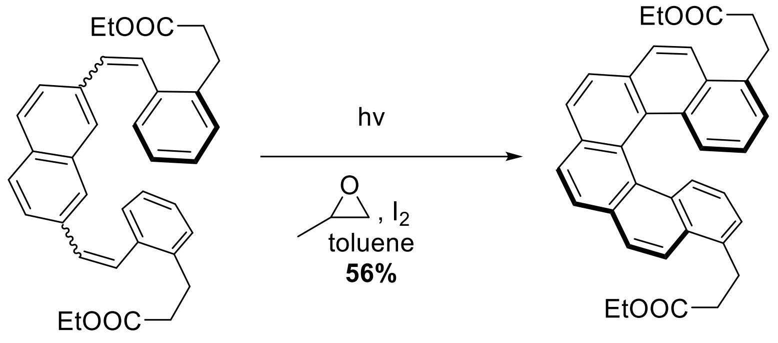

5 **6**

Diethyl 3,3'-(hexahelicene-9,16-diyl)dipropionate (6).

A solution of diethyl 3,3'-((naphthalene-2,7-diylbis(ethene-2,1-diyl))bis(2,1-phenylene))dipropionate (**5**; 964 mg, 1.00 mmol), methyl oxirane (64 mL, 0.91 mol) and iodine (1.01 g, 3.98 mmol) in toluene (1.2 L; *c*(**5**) = 1.5 mM) was purged with nitrogen for 30 min. The solution was irradiated in a continuous-flow photoreactor (flow rate = 0.035 mL/s) equipped with a glass cooling tube containing a 150 W medium-pressure mercury lamp from Heraeus. The collected solution was subsequently washed with aqueous $Na_2S_2O_3$ (sat.), water and brine, dried over anhydrous $MgSO_4$, filtered and concentrated under reduced pressure. The crude product was purified by flash column chromatography (SiO_2, CH_2Cl_2) to afford the desired product **6** as a yellow solid (540 mg, 56%).

^{1}H NMR (400 MHz, CD_2Cl_2, ppm): δ 8.18 (d, J = 8.9 Hz, 2H), 8.03 (d, J = 8.1 Hz, 2H), 8.00 (d, J = 8.9 Hz, 2H), 7.99 (d, J = 7.5 Hz, 2H), 7.42 (d, J = 8.6 Hz, 2H), 7.08 (d, J = 7.1 Hz, 2H), 6.54 (dd, J = 8.6, 7.1 Hz, 2H), 4.16 (q, J = 7.2 Hz, 4H), 3.54–3.39 (m, 4H), 2.85–2.71 (m, 4H), 1.26 (t, J = 7.1 Hz, 6H).

^{13}C NMR (101 MHz, CD_2Cl_2, ppm): δ 173.1, 136.7, 133.6, 131.1, 130.7, 130.1, 129.0, 127.4, 127.3, 127.0, 126.6, 126.2, 124.6, 124.5, 123.0, 60.8, 36.1, 29.0, 14.5.

HRMS (ESI) *m/z*: $[M + H]^+$ Calcd for $C_{36}H_{32}O_4$ 529.23734; Found 529.23748.

IR (cm^{-1}): 2977, 1720, 1438, 1202, 1167, 850, 503.

m.p. (°C): 132.9–134.7.

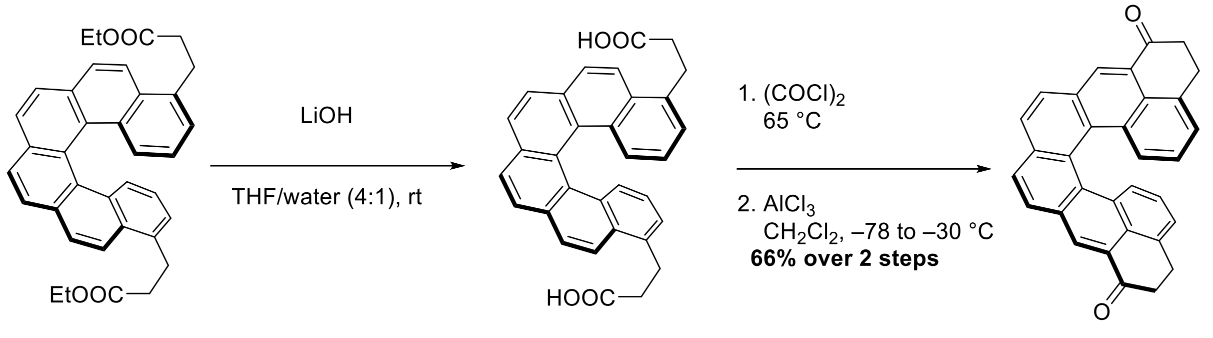

6 **S5** **7**

1,2,11,12-Tetrahydrobenzo[*no*]phenaleno[1,2-*a*]tetraphene-3,10-dione (7).

To a solution of diethyl 3,3'-(hexahelicene-9,16-diyl)dipropionate (**6**; 917 mg, 1.73 mmol) in THF/water (25.5 mL, 4:1), LiOH·6H_2O (290 mg, 6.92 mmol) was added and the resulting mixture was stirred at room temperature for 16 h before it was poured into aqueous HCl (10 mL, 1 M). The organic solvent was evaporated under reduced pressure, resulting in a precipitate, which was filtered, washed with water and freeze-dried to afford the diacid intermediate **S5** as a yellow solid (817 mg).

HRMS (ESI) *m*/*z*: [*M* + Na]$^+$ Calcd for $C_{32}H_{24}O_4$ 495.15668; Found 495.15689.

The next step was carried out under inert conditions. The diacid intermediate **S5** (98 mg, 0.21 mmol) was dissolved in oxalyl chloride (3.00 mL, 34.7 mmol) and the mixture was stirred at 65 °C for 2.5 h before it was taken to dryness using a gentle stream of nitrogen. The residue was redissolved in CH_2Cl_2 (10 mL) and cooled to –78 °C. $AlCl_3$ (166 mg, 1.25 mmol) was added and the reaction mixture was stirred at –78 °C for 1 h before it was allowed to warm to –30 °C over 4 h and, after quenching with aqueous HCl (1 mL, 2 M), to room temperature. The aqueous phase was extracted with CH_2Cl_2 (2 × 5 mL) and the combined organic layers were dried over anhydrous $MgSO_4$, filtered and concentrated under reduced pressure. The crude product was filtered through a short pad of silica and washed with CH_2Cl_2. The obtained residue was dissolved in the minimum amount of CH_2Cl_2, and MeOH was added to form a precipitate. The fine solid particles were separated with centrifuge and washed with MeOH to

afford the desired product **7** as a yellow solid with a green fluorescence (60 mg, 66%). Caution: The product decomposes in air over time and must be kept under an inert atmosphere.

^{1}H NMR (500 MHz, CD_2Cl_2, ppm): δ 8.70 (s, 2H), 8.21 (d, J = 8.1 Hz, 2H), 8.10 (d, J = 8.2 Hz, 2H), 7.46 (d, J = 8.5 Hz, 2H), 7.20 (dd, J = 7.0, 1.2 Hz, 2H), 6.71 (dd, J = 8.6, 7.0 Hz, 2H), 3.52 (ddd, J = 16.1, 6.4, 6.3 Hz, 2H), 3.41 (ddd, J = 15.9, 10.0, 5.7 Hz, 2H), 3.16 (ddd, J = 16.0, 9.9, 6.3 Hz, 2H), 3.01 (ddd, J = 15.4, 6.1, 6.0 Hz, 2H).

^{13}C NMR (126 MHz, CD_2Cl_2, ppm): δ 198.7, 136.0, 133.7, 132.2, 130.6, 130.3, 130.1, 129.5, 129.4, 127.9, 126.5, 126.3, 125.6, 125.4, 124.1, 39.1, 29.2.

HRMS (ESI) *m*/*z*: [*M* + H]$^+$ Calcd for $C_{32}H_{20}O_2$ 437.15361; Found 437.15375.

IR (cm^{-1}): 3058, 2918, 2849, 1683, 1579, 1238, 764.

m.p. (°C): 127.1 (decomposition).

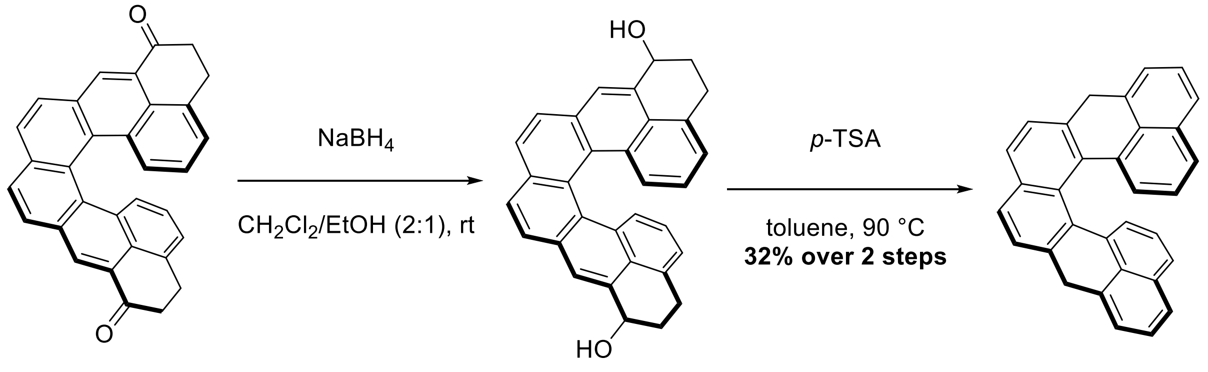

7 **S6** 2*H*-**1**

4,9-Dihydrobenzo[*no*]phenaleno[1,2-*a*]tetraphene (dihydro[8]cethrene, 2*H*-1).

To a solution of 1,2,11,12-tetrahydrobenzo[*no*]phenaleno[1,2-*a*]tetraphene-3,10-dione (**7**; 10 mg, 23 µmol) in CH_2Cl_2/EtOH (6 mL, 2:1), $NaBH_4$ (3.5 mg, 92 µmol) was added at 0 °C and the mixture was allowed to warm to room temperature. After stirring for 3 h, the mixture was poured into aqueous HCl (1 mL, 2 M) and extracted with CH_2Cl_2 (2 × 5 mL). The combined organic layers were dried over anhydrous $MgSO_4$, filtered and concentrated under reduced pressure to obtain intermediate **S6** as a light-yellow solid (10 mg).

HRMS (ESI) *m*/*z*: [*M* + Na]$^+$ Calcd for $C_{32}H_{24}O_2$ 463.16685; Found 463.16661.

The next step was carried out under inert conditions. To the solution of intermediate **S6** (10 mg, 23 μmol) in toluene (2 mL) deoxygenated by freeze-pump-thaw technique in three cycles, *p*-toluenesulfonic acid (8.7 mg, 46 μmol) was added and the mixture was stirred at 90 °C for 1 h before it was filtered through a pad of silica under nitrogen atmosphere and concentrated under reduced pressure to yield the desired product as a yellow solid (3.0 mg, 32%). The X-ray diffraction (XRD) analysis of single crystals of 2*H*-**1** (see section S4), obtained by slow evaporation of solvent from a CD_2Cl_2 solution under a nitrogen stream at room temperature, supports the structure of 2*H*-**1** with a C_2 symmetry.

^{1}H NMR (500 MHz, CD_2Cl_2, ppm): δ 7.79 (d, *J* = 8.0 Hz, 2H), 7.63 (dd, *J* = 4.8, 4.6 Hz, 2H), 7.47 (d, *J* = 7.9 Hz, 2H), 7.46–7.44 (m, 4H), 7.34 (d, *J* = 8.1 Hz, 2H), 6.87 (d, *J* = 7.3 Hz, 2H), 6.36 (dd, *J* = 7.8, 7.7 Hz, 2H), 4.77 (d, *J* = 22.7 Hz, 2H), 4.71 (d, *J* = 22.6 Hz, 2H).

^{13}C NMR (126 MHz, CD_2Cl_2, ppm): δ 136.6, 134.8, 133.6, 133.5, 131.5, 130.5, 130.0, 128.2, 127.4, 126.33, 126.29, 125.9, 125.8, 125.4, 125.1, 123.9, 36.0.

HRMS (EI) *m*/*z*: [*M*]$^+$ Calcd for $C_{32}H_{20}$ 404.15595; Found 404.15550.

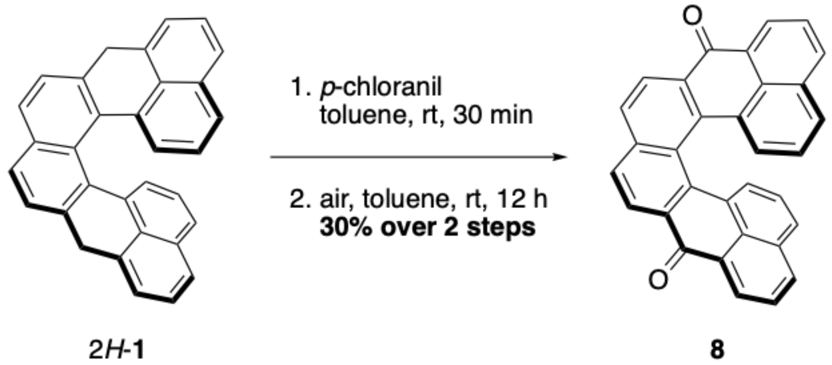

Benzo[*no*]phenaleno[1,2-*a*]tetraphene-4,9-dione (8).

The first part of the reaction was carried out under inert conditions. To a solution of dihydro[8]cethrene (2*H*-**1**; 11.6 mg, 28.8 μmol) in toluene (40 mL, deoxygenated by freeze-pump-thaw technique in three cycles), *p*-chloranil (14.2 mg, 57.6 μmol) was added and the

mixture was stirred at room temperature for 30 min, leading to the formation of a precipitate, before being exposed to air and stirred for an additional 12 h, during which the suspension gradually turned into a solution. The reaction mixture was then concentrated under reduced pressure and the crude was purified by flash column chromatography (SiO_2, CH_2Cl_2) to yield the product as a yellow solid (3.8 mg, 30%).

^{1}H NMR (400 MHz, CD_2Cl_2, ppm): δ 8.80 (dd, J = 7.3, 1.3 Hz, 2H), 8.66 (d, J = 8.2 Hz, 2H), 8.24 (dd, J = 8.0, 1.3 Hz, 2H), 8.07 (d, J = 8.3 Hz, 2H), 7.85 (dd, J = 7.8, 7.5 Hz, 2H), 7.69 (d, J = 8.0 Hz, 2H), 7.56 (dd, J = 7.4, 1.0 Hz, 2H), 6.75 (dd, J = 7.9, 7.7 Hz, 2H).

^{13}C NMR (126 MHz, CD_2Cl_2, ppm): δ 183.6, 140.8, 138.9, 135.8, 133.8, 133.0, 131.9, 123.0, 129.8, 129.0, 128.9, 127.4, 127.1, 127.0, 126.2, 125.7, 125.5.

HRMS (ESI) *m*/*z*: $[M + H]^+$ Calcd for $C_{33}H_{16}O_2$ 433.12231; Found 433.12238.

2 Nuclear magnetic resonance (NMR) spectroscopy

All ^{1}H, ^{13}C and ^{31}P NMR spectra, including 2D NMR spectra (COSY, NOESY, HSQC, HMBC) were recorded at a constant temperature of 298 K on Bruker 400 MHz, Bruker 500 MHz or Bruker 600 MHz. Standard pulse sequences were used, and the data were processed using twofold zero-filling in the indirect dimension for all 2D experiments. Chemical shifts (δ) are reported in parts per million (ppm) relative to the solvent residual peak (^{1}H and ^{13}C NMR, respectively): $CDCl_3$ (δ = 7.26 and 77.16 ppm) and CD_2Cl_2 (δ = 5.32 and 53.84 ppm)[4].

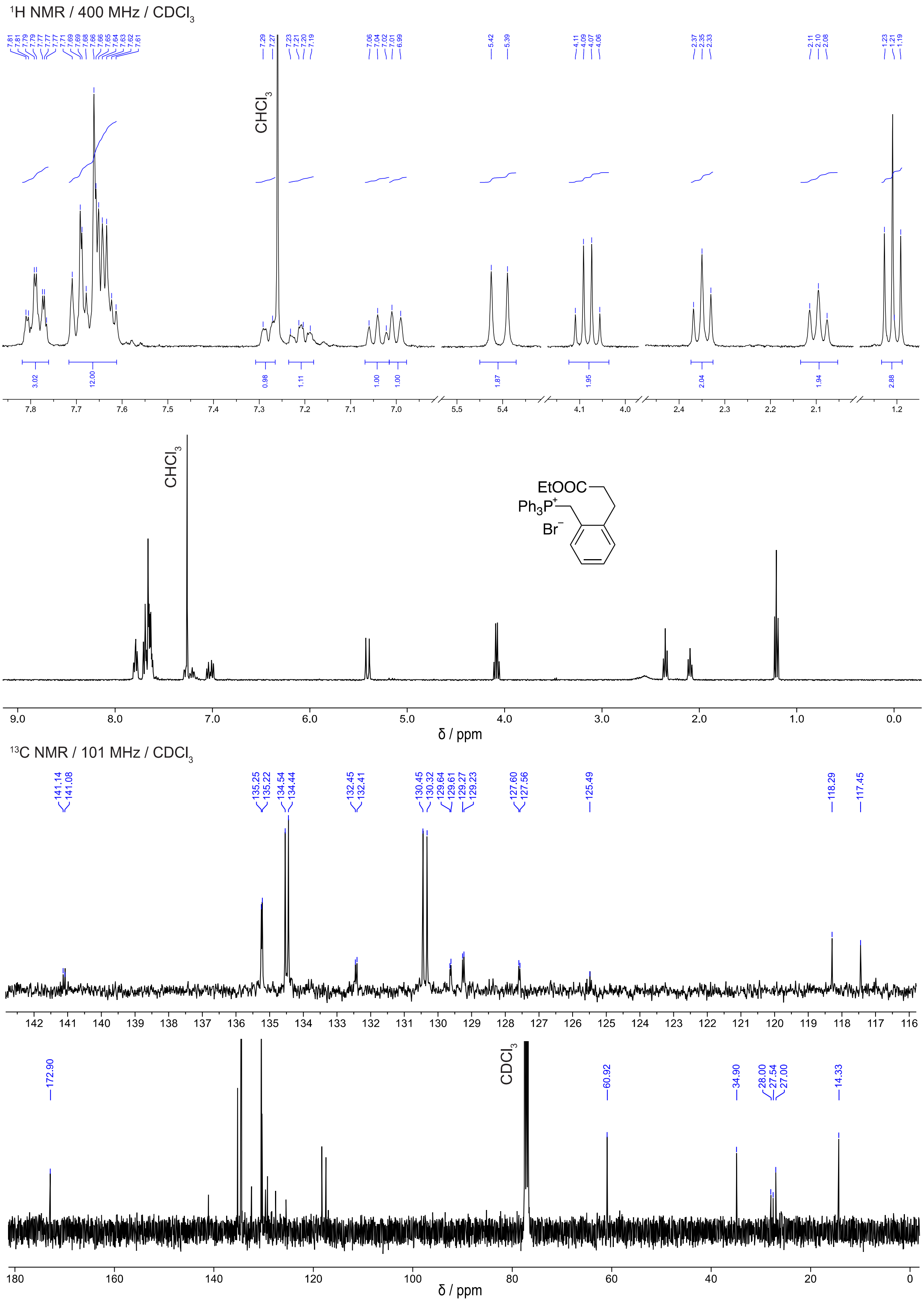

Figure S1. ^{1}H and ^{13}C NMR copies for (2-(3-ethoxy-3-oxopropyl)benzyl)triphenylphosphonium bromide (**4**).

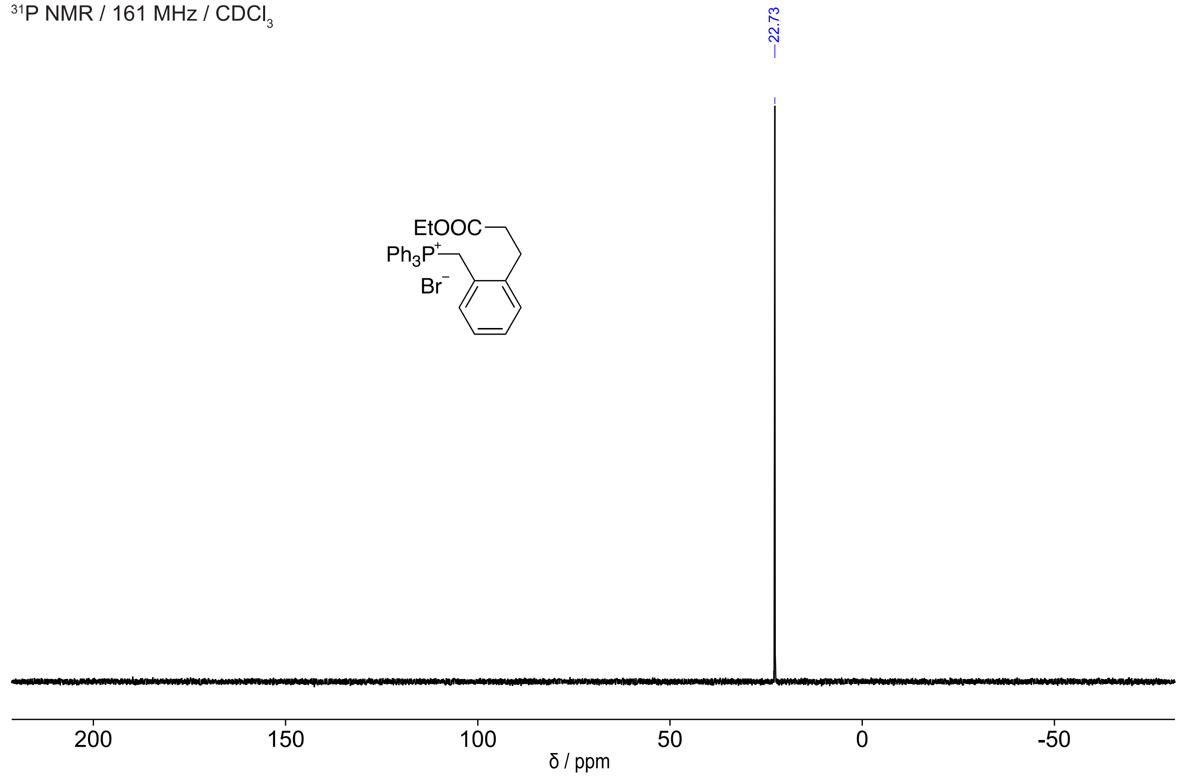

Figure S2. ^{31}P NMR copy for (2-(3-ethoxy-3-oxopropyl)benzyl)triphenylphosphonium bromide (**4**).

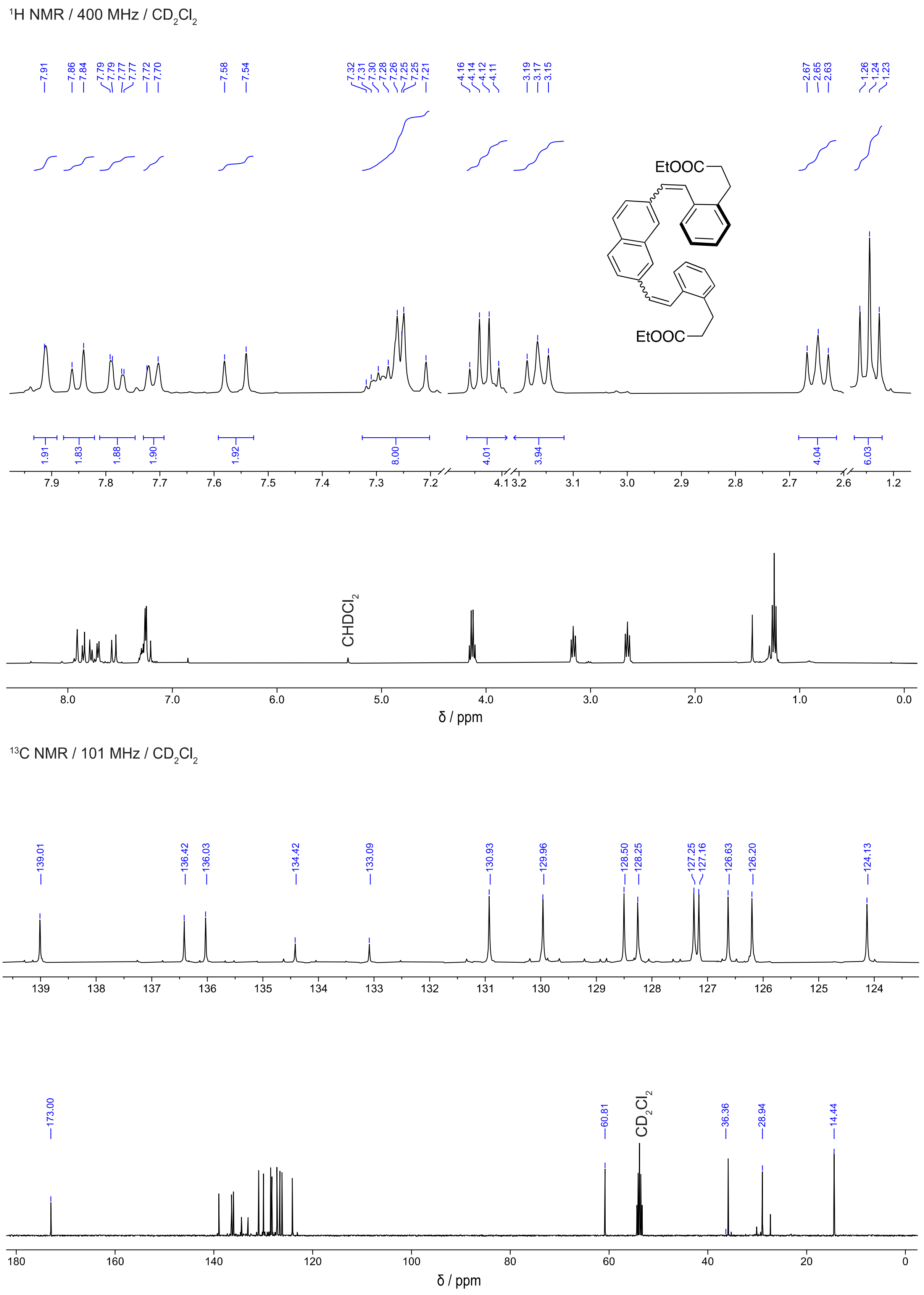

Figure S3. ^{1}H and ^{13}C NMR copies for diethyl 3,3'-((naphthalene-2,7-diylbis(ethene-2,1-diyl))bis(2,1-phenylene))dipropionate (**5**).

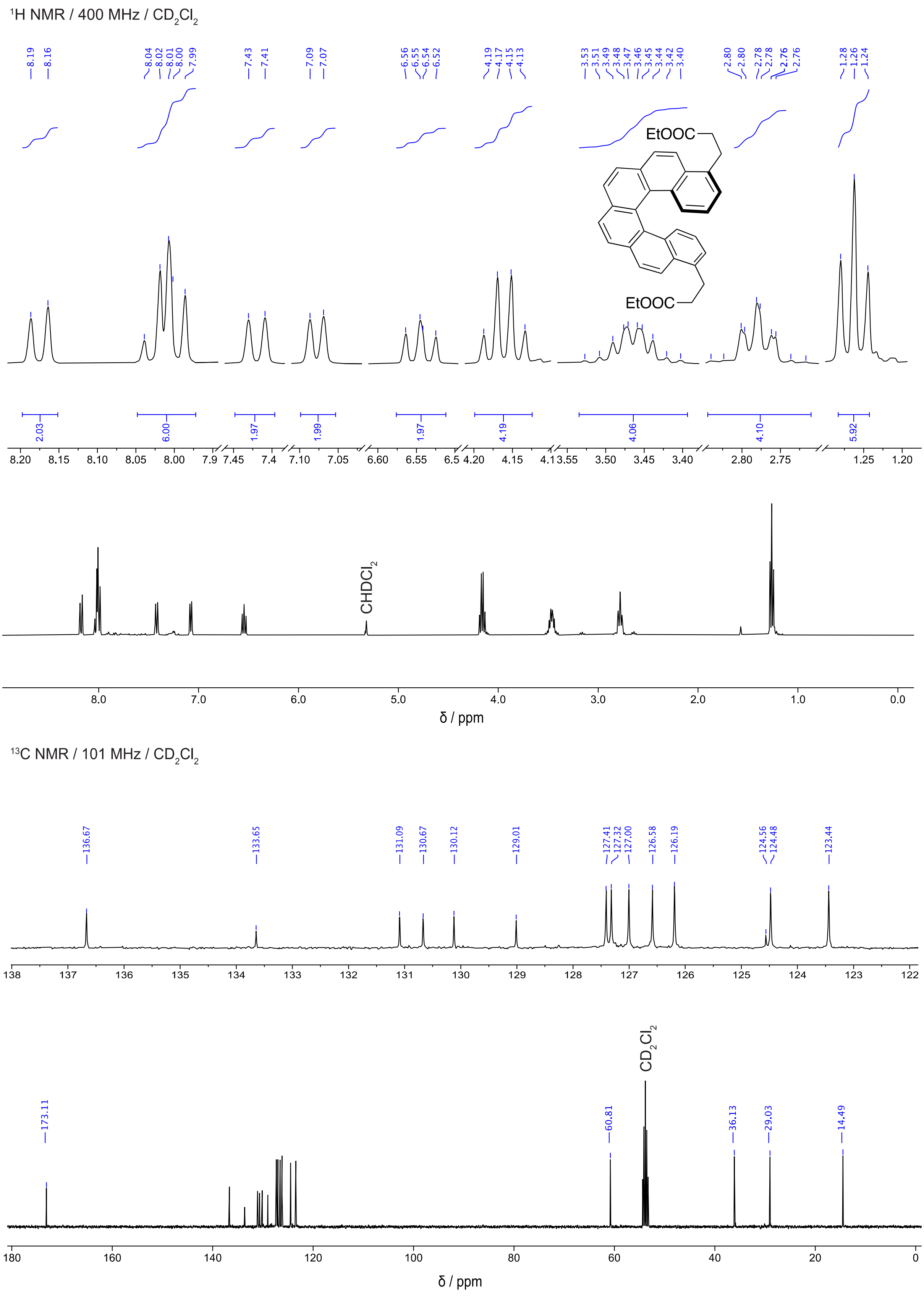

Figure S4. ^{1}H and ^{13}C NMR copies for diethyl 3,3'-(hexahelicene-9,16-diyl)dipropionate (**6**).

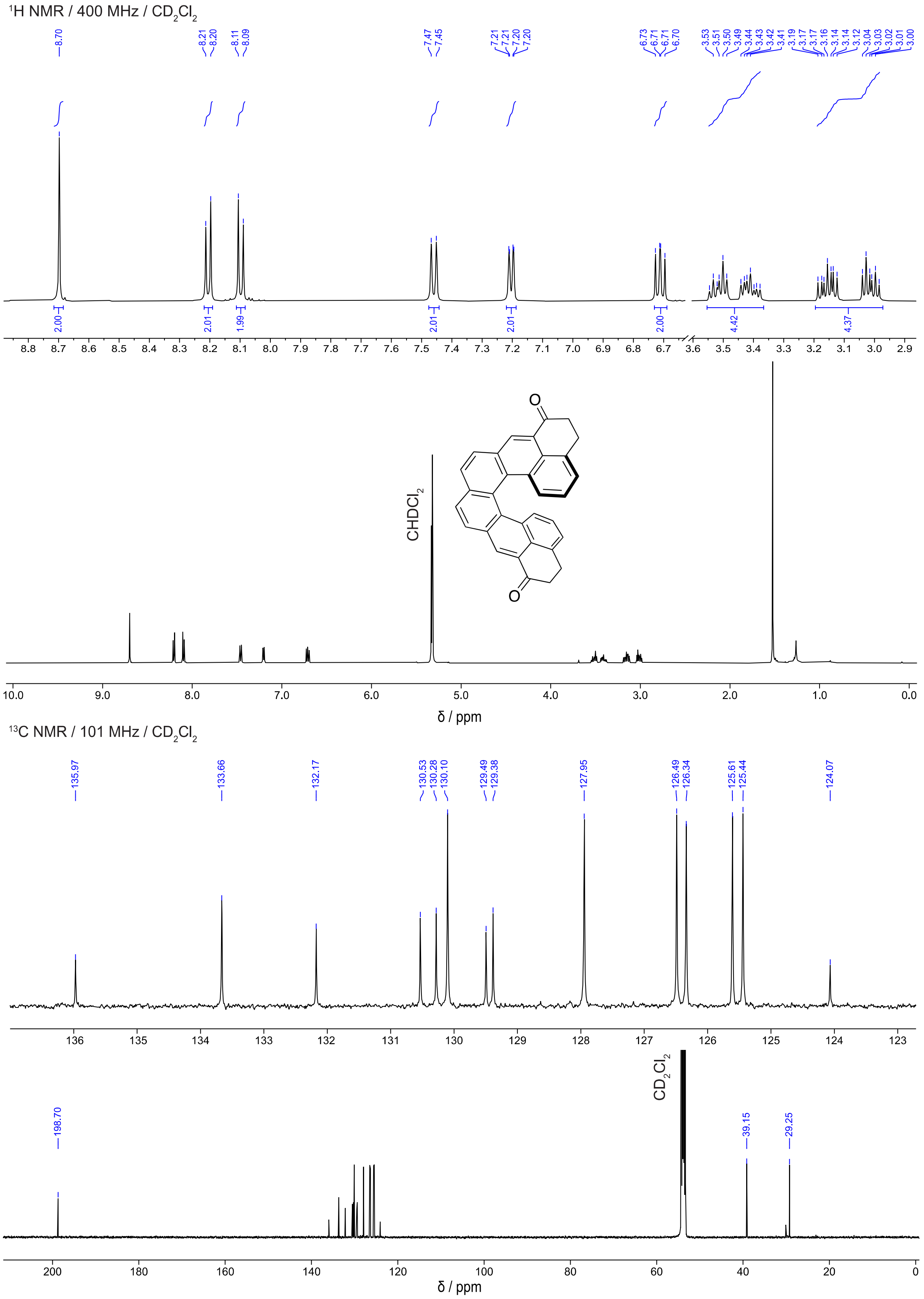

Figure S5. ^{1}H and ^{13}C NMR copies for 1,2,11,12-tetrahydrobenzo[*no*]phenaleno[1,2-*a*]tetraphene-3,10-dione (**7**).

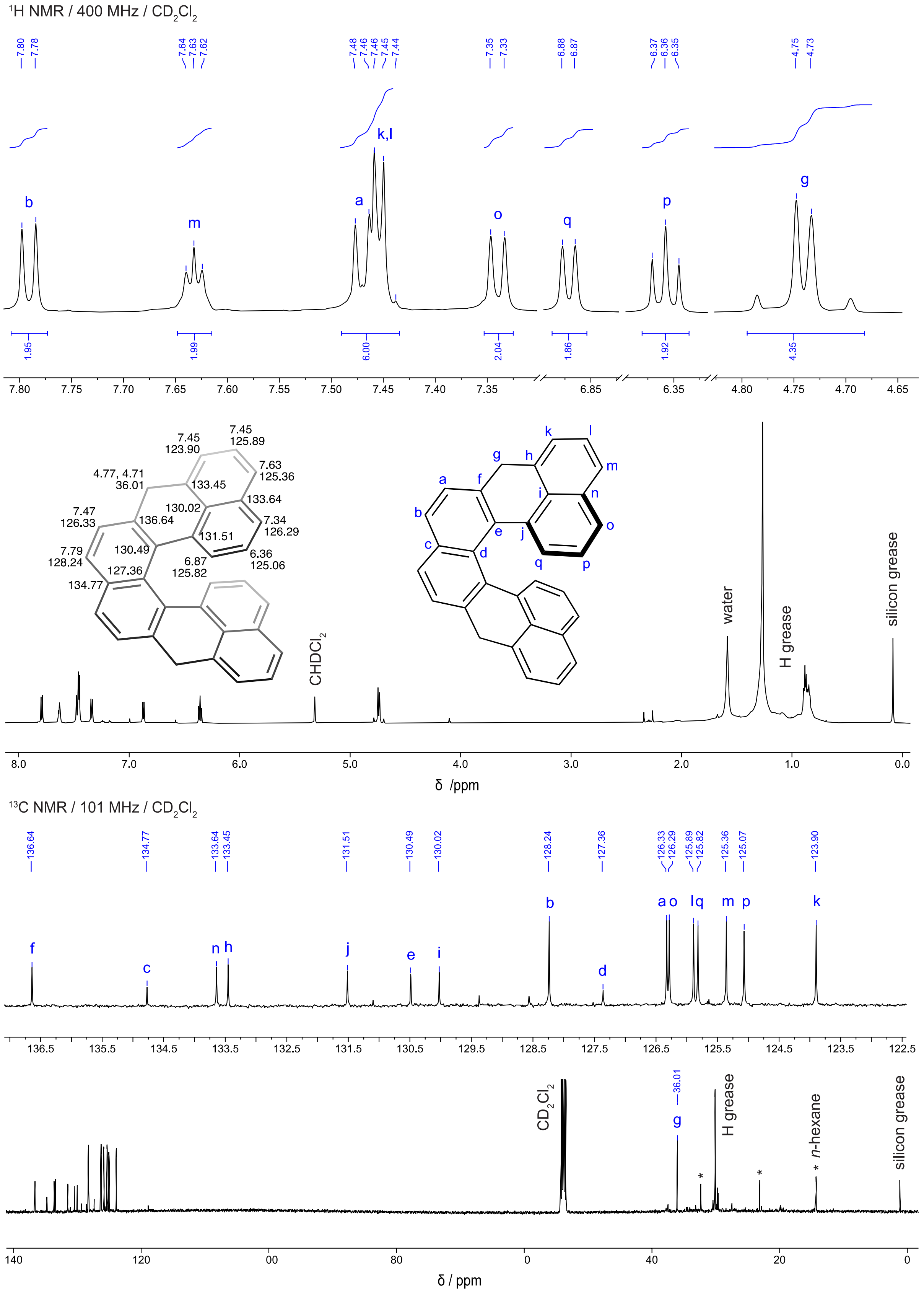

Figure S6. ^{1}H and ^{13}C NMR copies of dihydro[8]cethrene (2*H*-**1**).

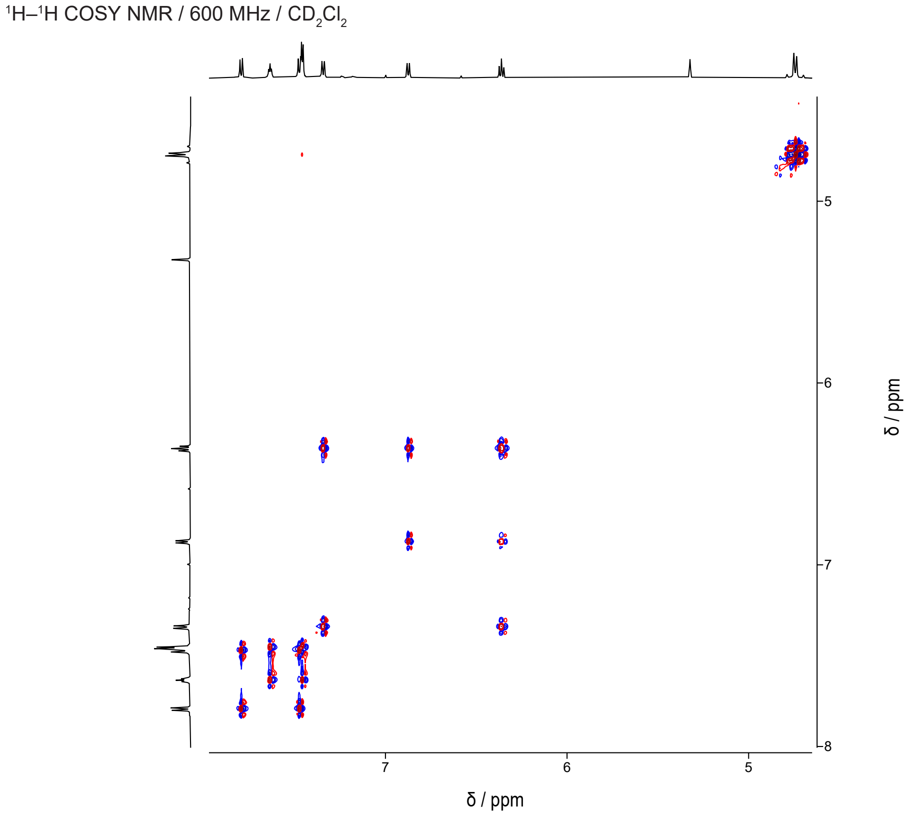

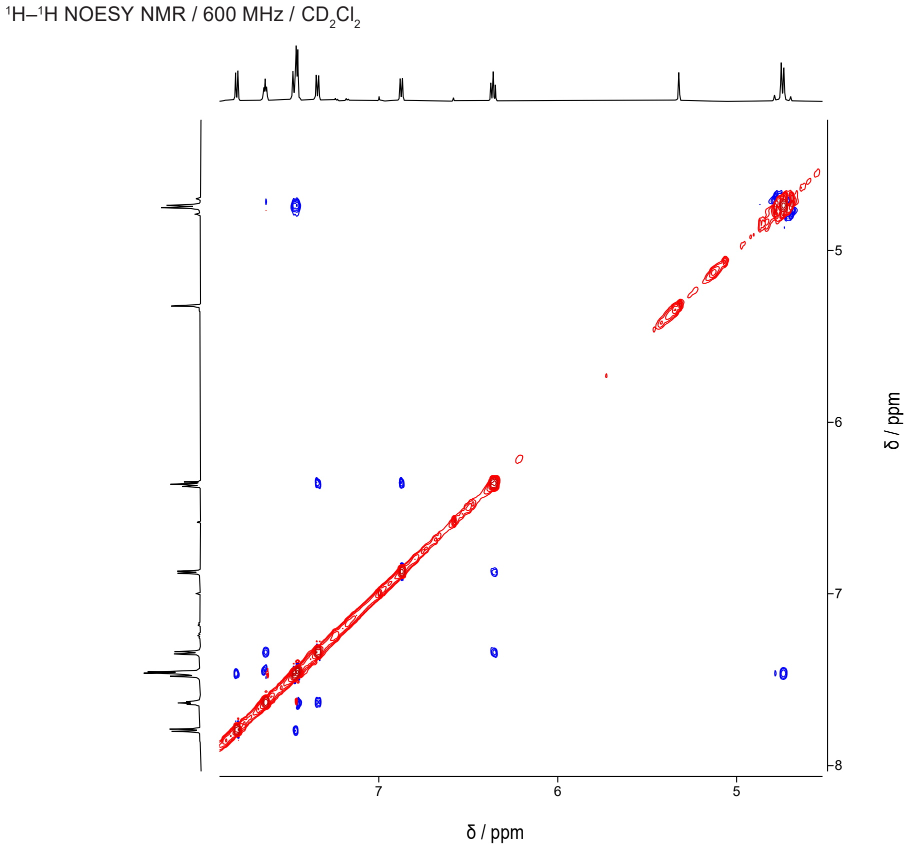

Figure S7. COSY and NOESY NMR copies of dihydro[8]cethrene (2*H*-**1**).

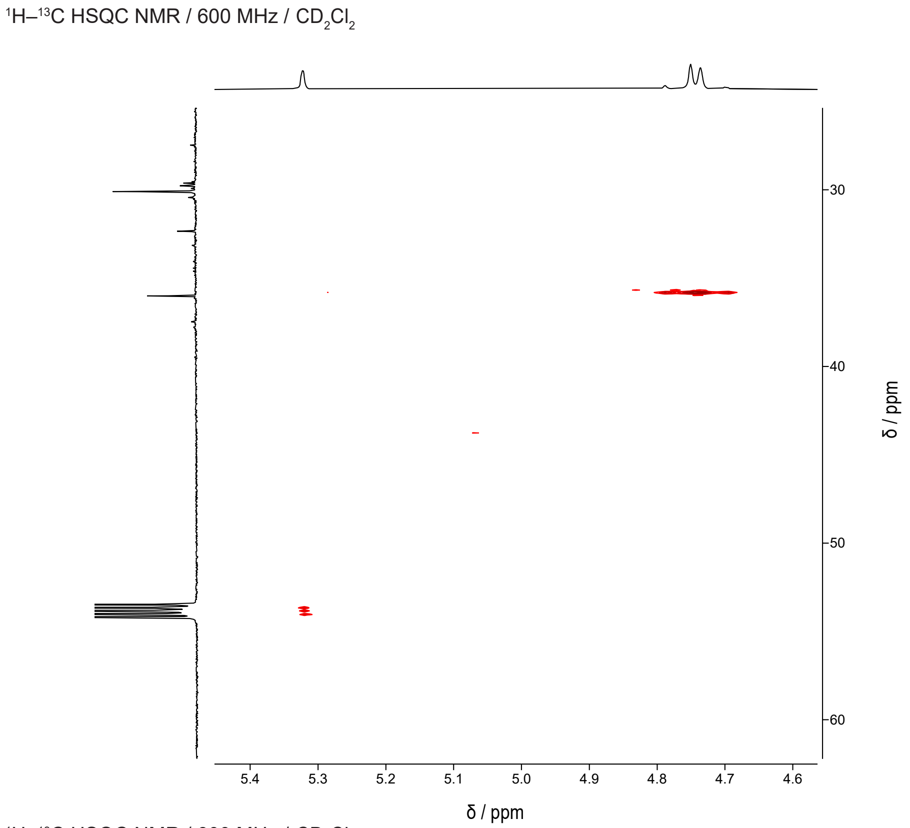

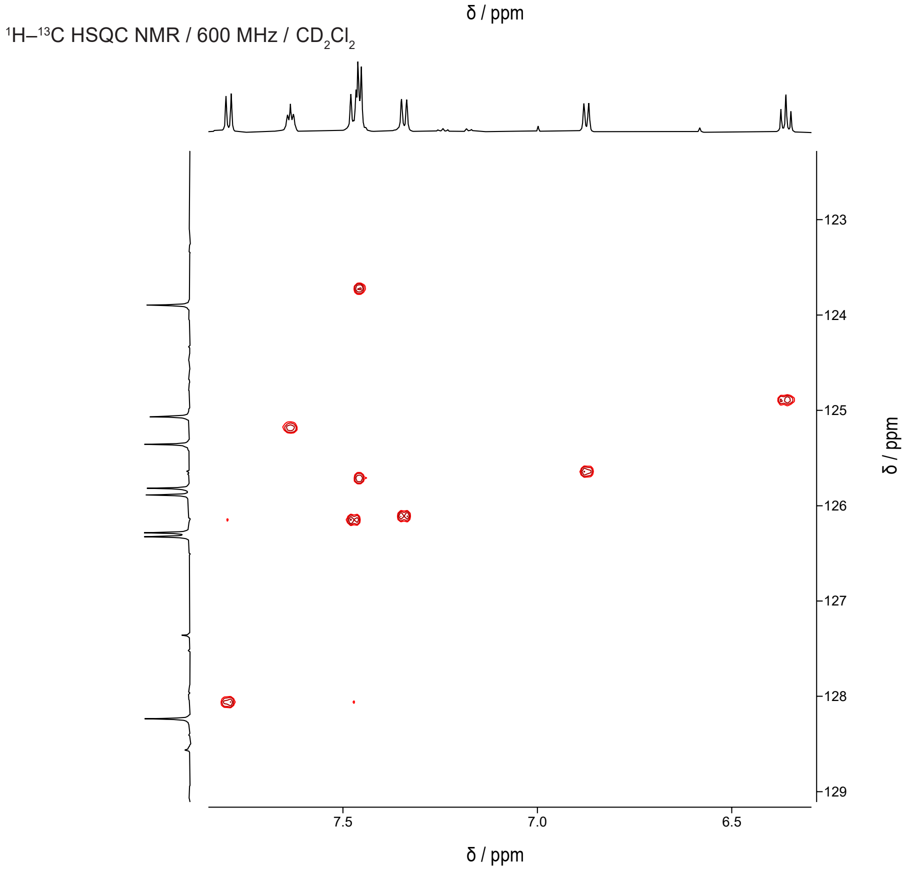

Figure S8. HSQC NMR copies of dihydro[8]cethrene (2*H*-**1**).

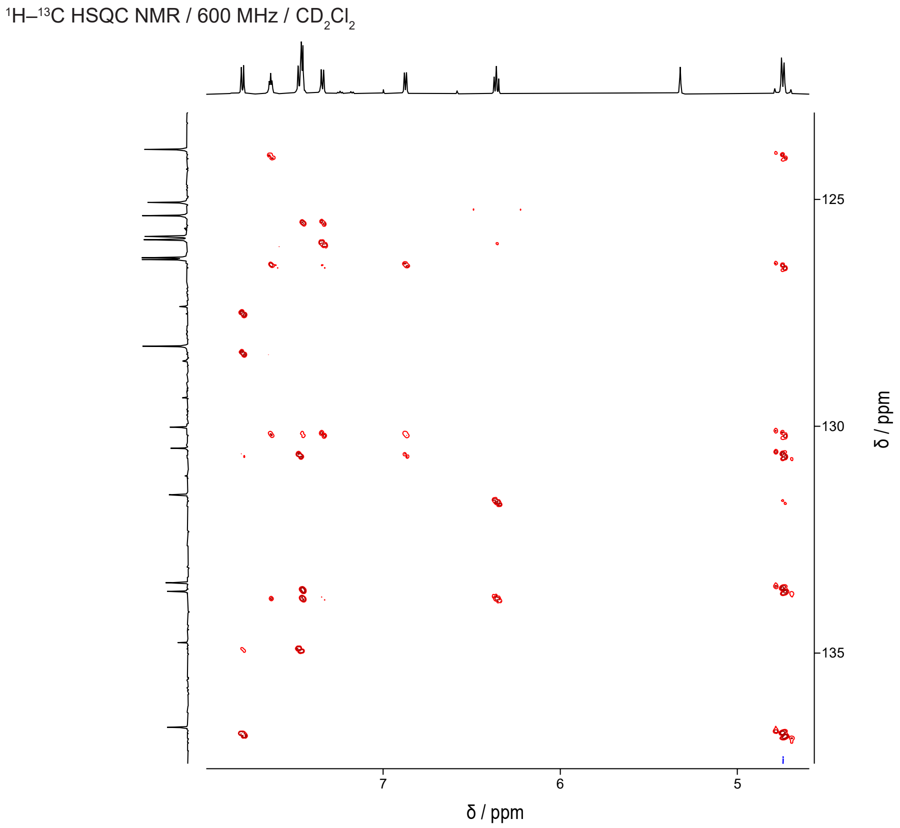

Figure S9. HMBC NMR copy of dihydro[8]cethrene (2*H*-**1**).

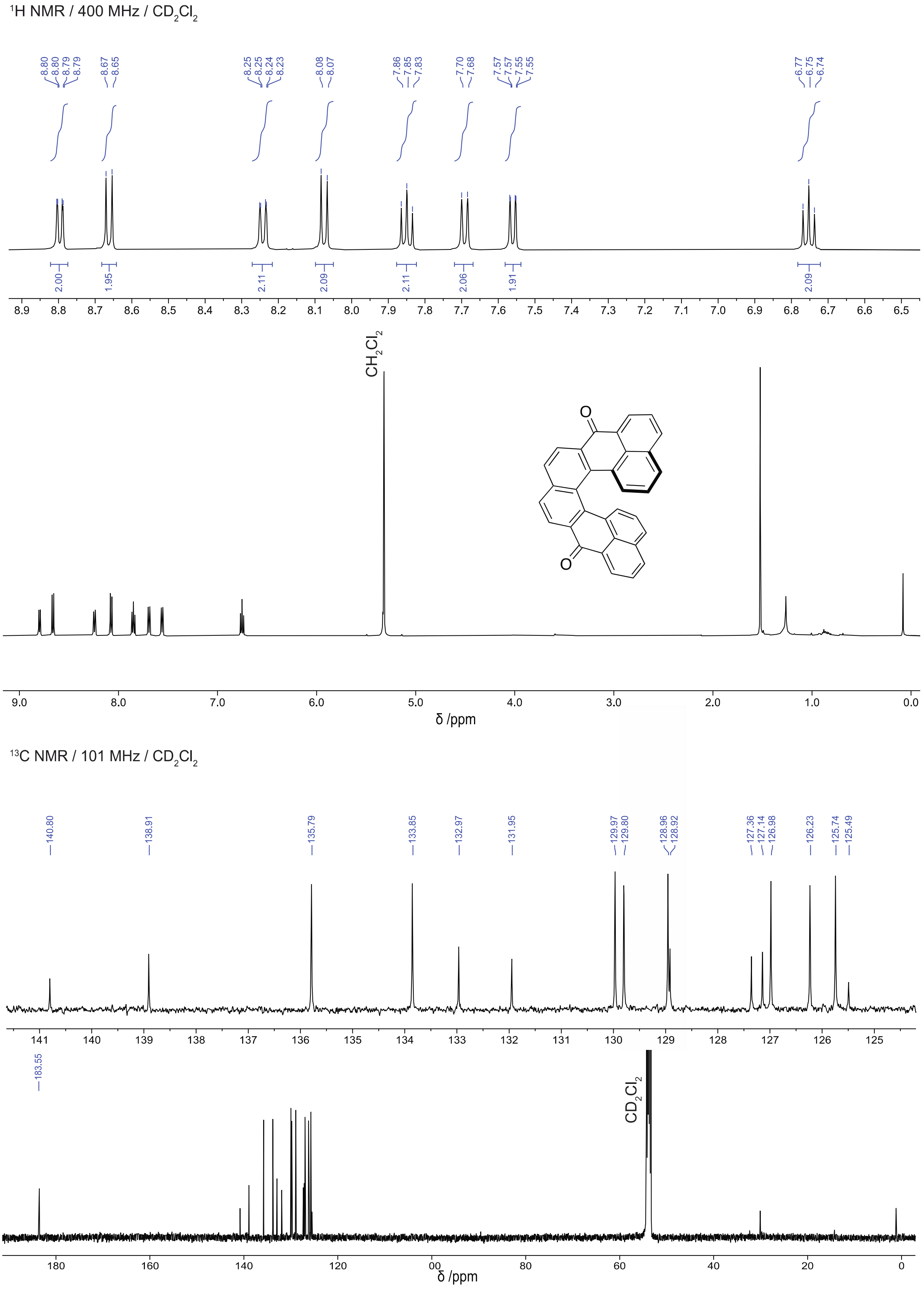

Figure S10. ^1H and ^{13}C NMR copies of benzo[*no*]phenaleno[1,2-*a*]tetraphene-4,9-dione (**8**).

3 Mass spectrometry

Electron ionisation (EI) high-resolution mass spectrometry (HRMS): double-focusing (BE geometry) magnetic sector mass spectrometer DFS (ThermoFisher Scientific, Bremen, Germany); solid probe inlet; EI at 70 eV; source temperature 200 °C; acceleration voltage 5 kV; electric scan mode; mass range 300–350 *m*/*z* at 10’000 resolution (10% valley definition) and scan rate of 100–200 s per decade; mass accuracy ≤ 2 ppm after calibration with perfluorokerosene (PFK, Fluorochem, Derbyshire, UK).

Electrospray ionisation (ESI) high-resolution mass spectra (HRMS) were recorded on a timsTOF Pro TIMS-QTOF-MS instrument (Bruker Daltonics GmbH, Bremen, Germany). The samples were dissolved (e.g., in MeOH) at a concentration of ca. 50 µg mL^{-1} and analysed via continuous flow injection (2 µL min^{-1}). The mass spectrometer was operated in the positive (or negative) electrospray ionisation mode at 4’000 V (–4’000 V) capillary voltage and –500 V (500 V) endplate offset with a N_2 nebuliser pressure of 0.4 bar and a dry gas flow of 4 L min^{-1} at 180 °C. Mass spectra were acquired in a mass range from *m*/*z* 50 to 2’000 at ca. 20’000 resolution (*m*/*z* 622) and at 1.0 Hz rate. The mass analyser was calibrated between *m*/*z* 118 and 2’721 using an Agilent ESI-L low concentration tuning mix solution (Agilent, USA) at a resolution of 20’000 giving a mass accuracy below 2 ppm. All solvent used were purchased in best LC-MS quality.

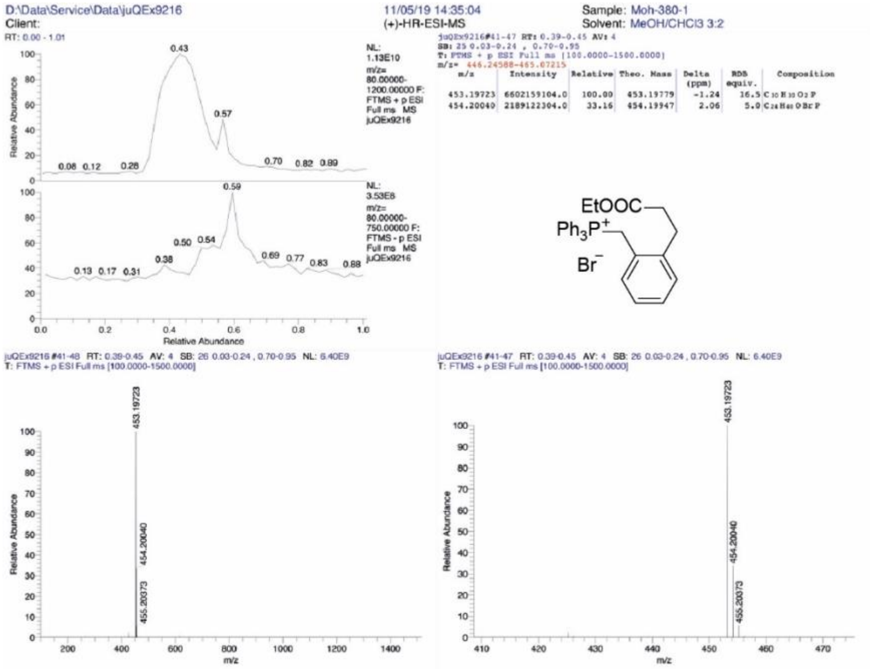

Figure S11. HRMS (ESI): (2-(3-ethoxy-3-oxopropyl)benzyl)triphenylphosphonium bromide (**4**).

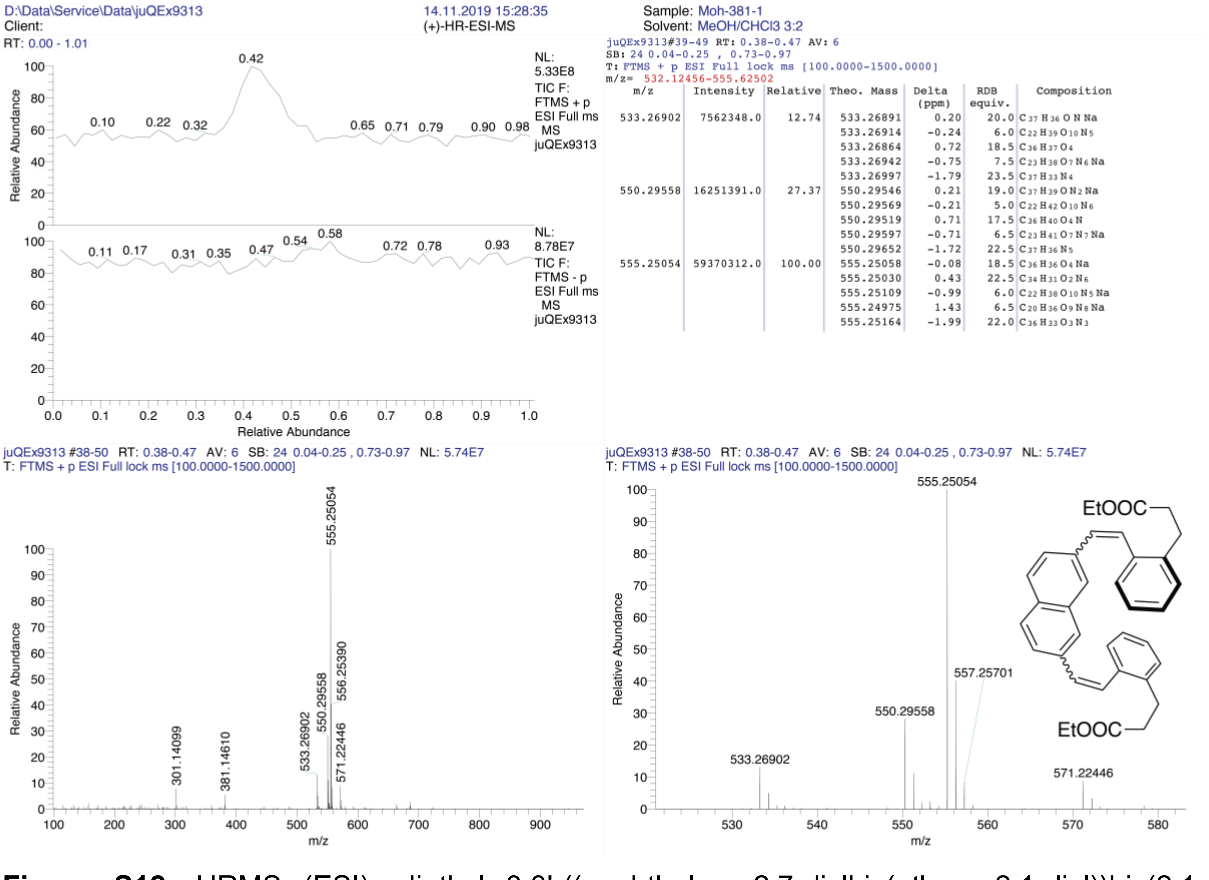

Figure S12. HRMS (ESI): diethyl 3,3'-((naphthalene-2,7-diylbis(ethene-2,1-diyl))bis(2,1-phenylene))dipropionate (**5**).

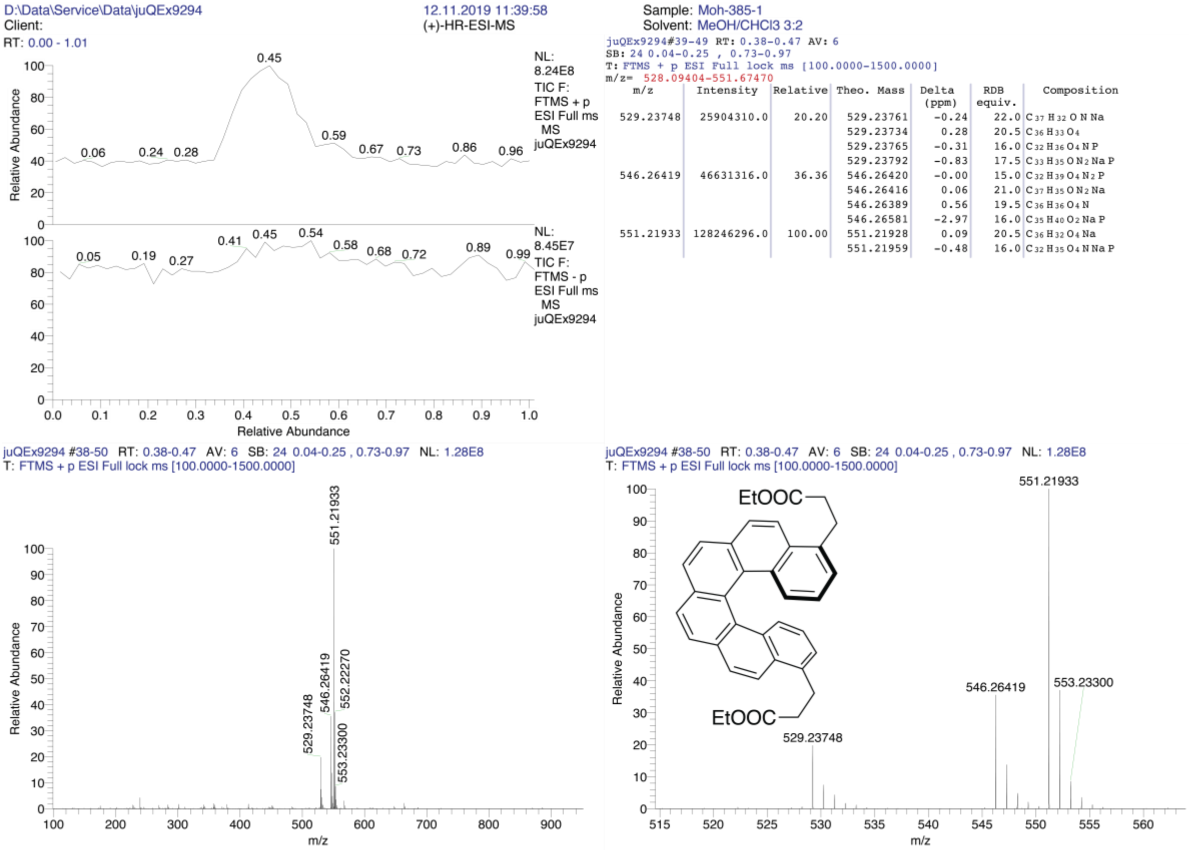

Sample: Moh-385-1
Solvent: MeOH/CHCl3 3:2

juQEx9294#39-49 RT: 0.38-0.47 AV: 6
SB: 24 0.04-0.25 , 0.73-0.97
T: FTMS + p ESI Full lock ms [100.0000-1500.0000]
m/z= 528.09404-551.67470

m/z	Intensity	Relative	Theo. Mass	Delta (ppm)	RDB equiv.	Composition
529.23748	25904310.0	20.20	529.23761	-0.24	22.0	$C_{37}H_{32}ONNa$
			529.23734	0.28	20.5	$C_{36}H_{33}O_4$
			529.23765	-0.31	16.0	$C_{32}H_{36}O_4NP$
			529.23792	-0.83	17.5	$C_{33}H_{35}ON_2NaP$
546.26419	46631316.0	36.36	546.26420	-0.00	15.0	$C_{32}H_{39}O_4N_2P$
			546.26416	0.06	21.0	$C_{37}H_{35}ON_2Na$
			546.26389	0.56	19.5	$C_{36}H_{36}O_4N$
			546.26581	-2.97	16.0	$C_{35}H_{40}O_2NaP$
551.21933	128246296.0	100.00	551.21928	0.09	20.5	$C_{36}H_{32}O_4Na$
			551.21959	-0.48	16.0	$C_{32}H_{35}O_4NNaP$

Figure S13. HRMS (ESI): diethyl 3,3'-(hexahelicene-9,16-diyl)dipropionate (**6**).

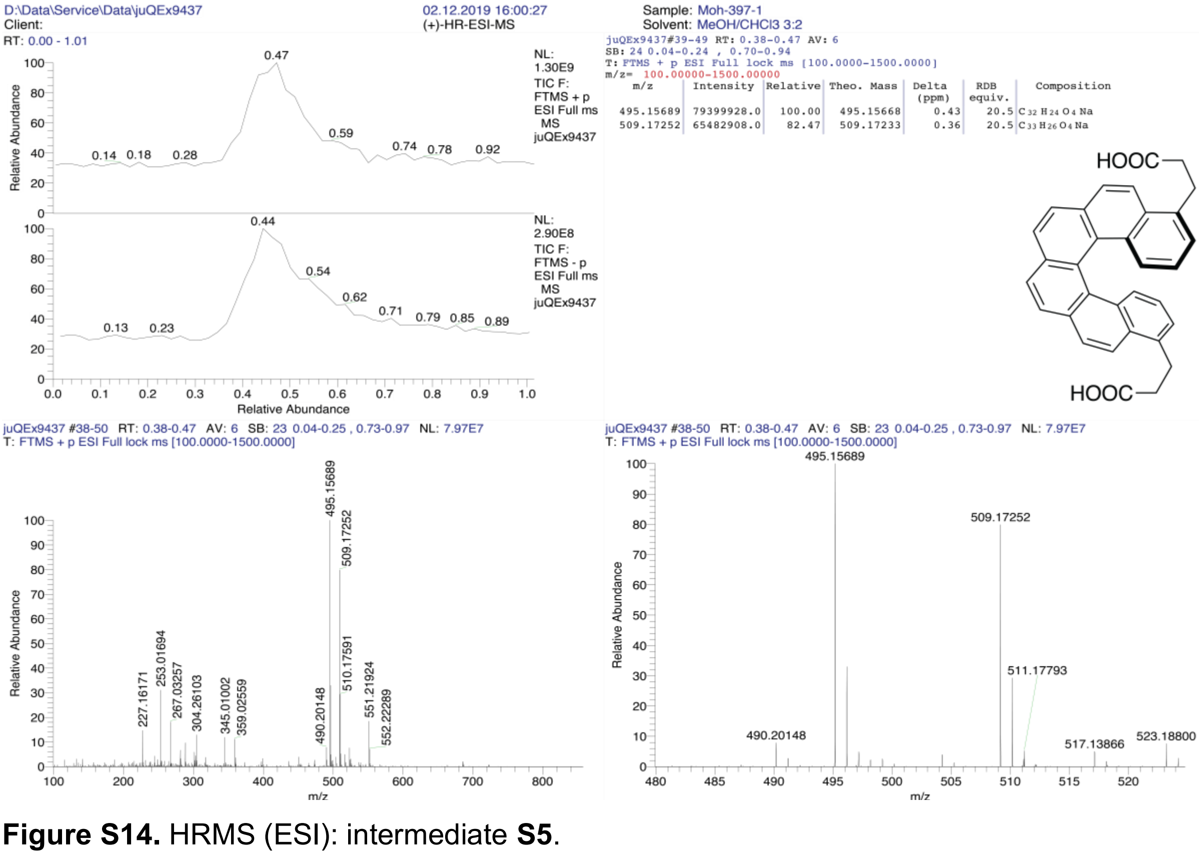

Sample: Moh-397-1
Solvent: MeOH/CHCl3 3:2

juQEx9437#39-49 RT: 0.38-0.47 AV: 6
SB: 24 0.04-0.24 , 0.70-0.94
T: FTMS + p ESI Full lock ms [100.0000-1500.0000]
m/z= 100.00000-1500.00000

m/z	Intensity	Relative	Theo. Mass	Delta (ppm)	RDB equiv.	Composition
495.15689	79399928.0	100.00	495.15668	0.43	20.5	$C_{32}H_{24}O_4Na$
509.17252	65482908.0	82.47	509.17233	0.36	20.5	$C_{33}H_{26}O_4Na$

Figure S14. HRMS (ESI): intermediate **S5**.

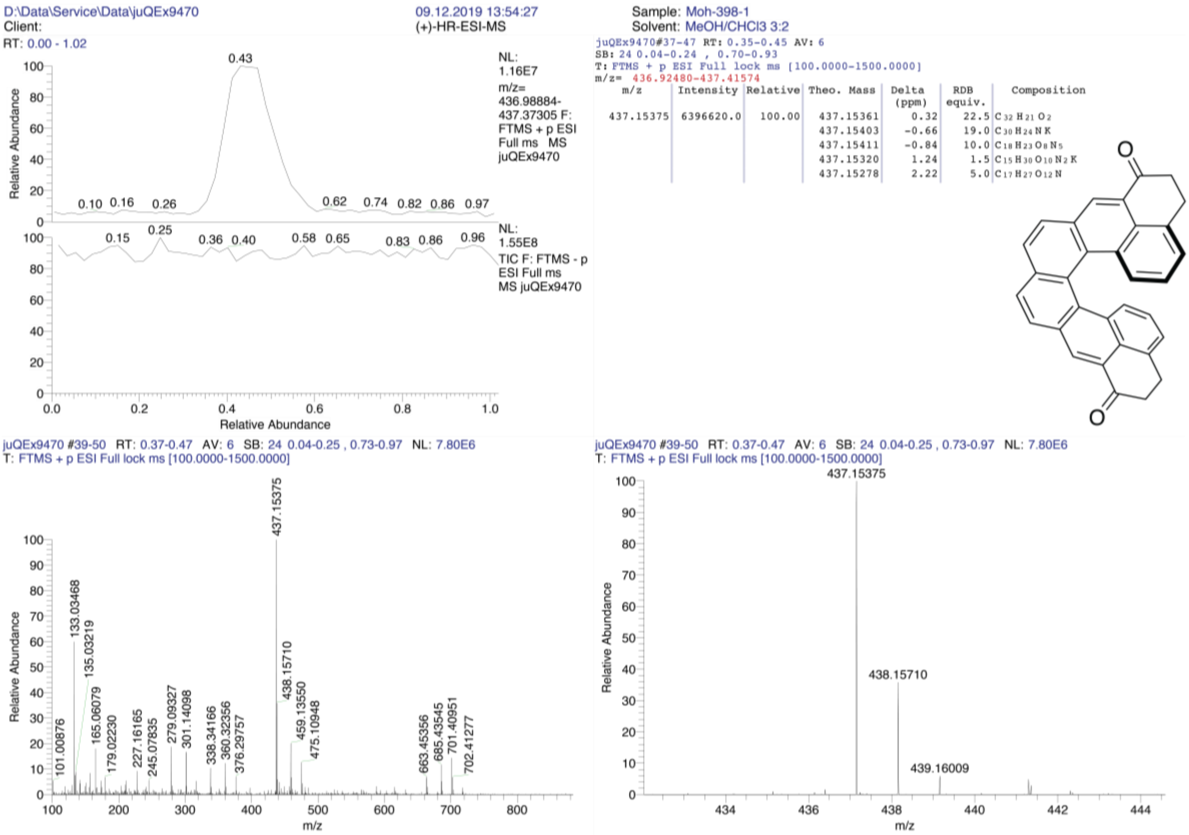

Figure S15. HRMS (ESI): 1,2,11,12-tetrahydrobenzo[*no*]phenaleno[1,2-*a*]tetraphene-3,10-dione (**7**).

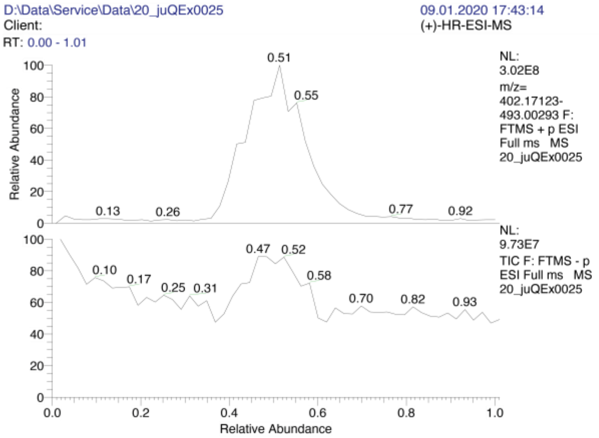

Sample: Moh-412-1
Solvent: MeOH/CHCl3 3:2

20_juQEx0025#39-49 RT: 0.38-0.48 AV: 6
SB: 24 0.04-0.25 , 0.73-0.97
T: FTMS + p ESI Full lock ms [100.0000-1500.0000]
m/z= 420.63308-483.28400

m/z	Intensity	Relative	Theo. Mass	Delta (ppm)	RDB equiv.	Composition
423.17413	45217088.0	100.00	423.17434	-0.49	21.5	$C_{32}H_{23}O$
424.17747	15598297.0	34.50	424.17751	-0.10	10.0	$C_{25}H_{30}O_2KNa$
437.19020	15618479.0	34.54	437.18999	0.47	21.5	$C_{33}H_{25}O$
463.16661	13332139.0	29.48	463.16685	-0.51	20.5	$C_{32}H_{24}O_2Na$
			463.16700	-0.84	15.5	$C_{29}H_{28}O_3K$
			463.16728	-1.43	17.0	$C_{30}H_{27}NKNa$
479.14080	5559047.0	12.29	479.14079	0.03	20.5	$C_{32}H_{24}O_2K$
			479.14064	0.34	25.5	$C_{35}H_{20}ONa$

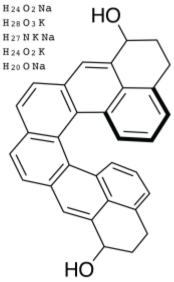

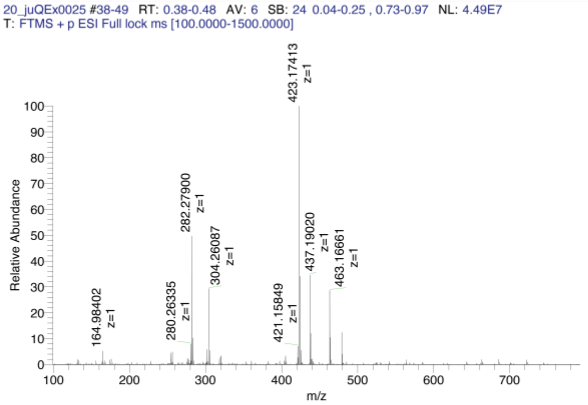

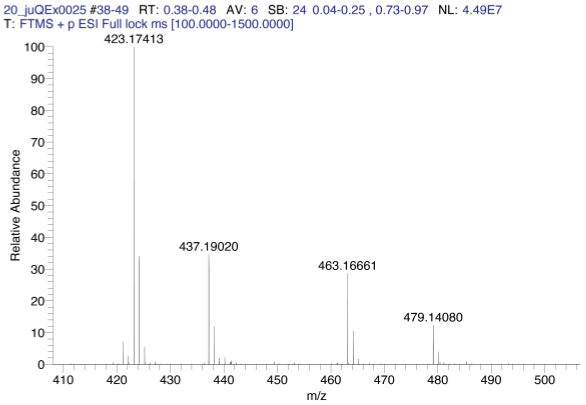

HR-EI-Report (Thermo DFS)

Sample:

Client:

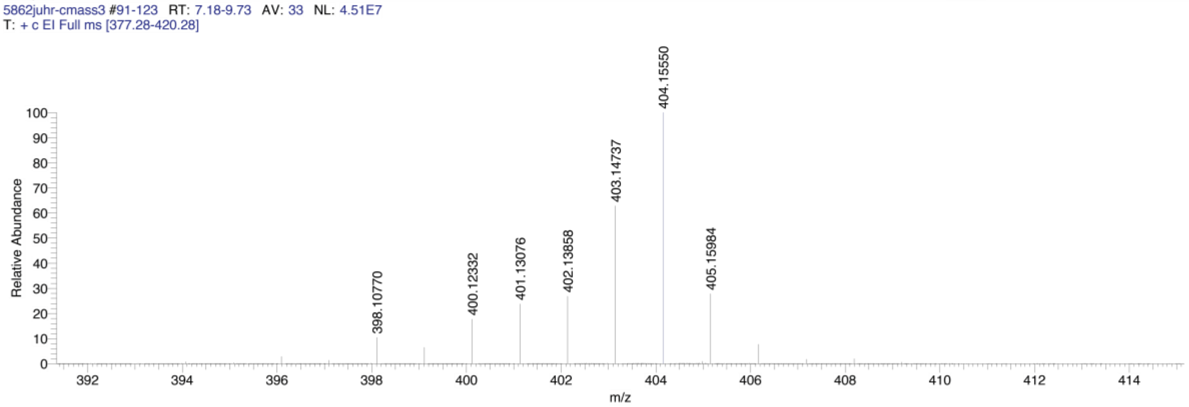

5862juhr-cmass3#91-123 RT: 7.18-9.73 AV: 33
T: + c EI Full ms [377.28-420.28]
m/z= 397.05315-405.79389

m/z	Intensity	Relative	Theo. Mass	Delta (ppm)	Composition
398.10770	4701150.3	10.43	398.10900	-3.27	$C_{32}H_{14}$
399.11317	2906518.2	6.45	399.11683	-9.17	$C_{32}H_{15}$
400.12332	7964862.8	17.67	400.12465	-3.32	$C_{32}H_{16}$
401.13076	10722565.8	23.79	401.13248	-4.27	$C_{32}H_{17}$
402.13858	12104601.2	26.85	402.14030	-4.28	$C_{32}H_{18}$
403.14737	28307128.2	62.80	403.14813	-1.88	$C_{32}H_{19}$
404.15550	45076619.0	100.00	404.15595	-1.13	$C_{32}H_{20}$
405.15984	12526845.7	27.79	405.16378	-9.72	$C_{32}H_{21}$

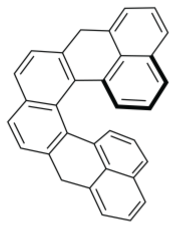

Figure S16. HRMS: intermediate **S6** (ESI, top) and dihydro[8]cethrene (2*H*-**1**; EI, bottom).

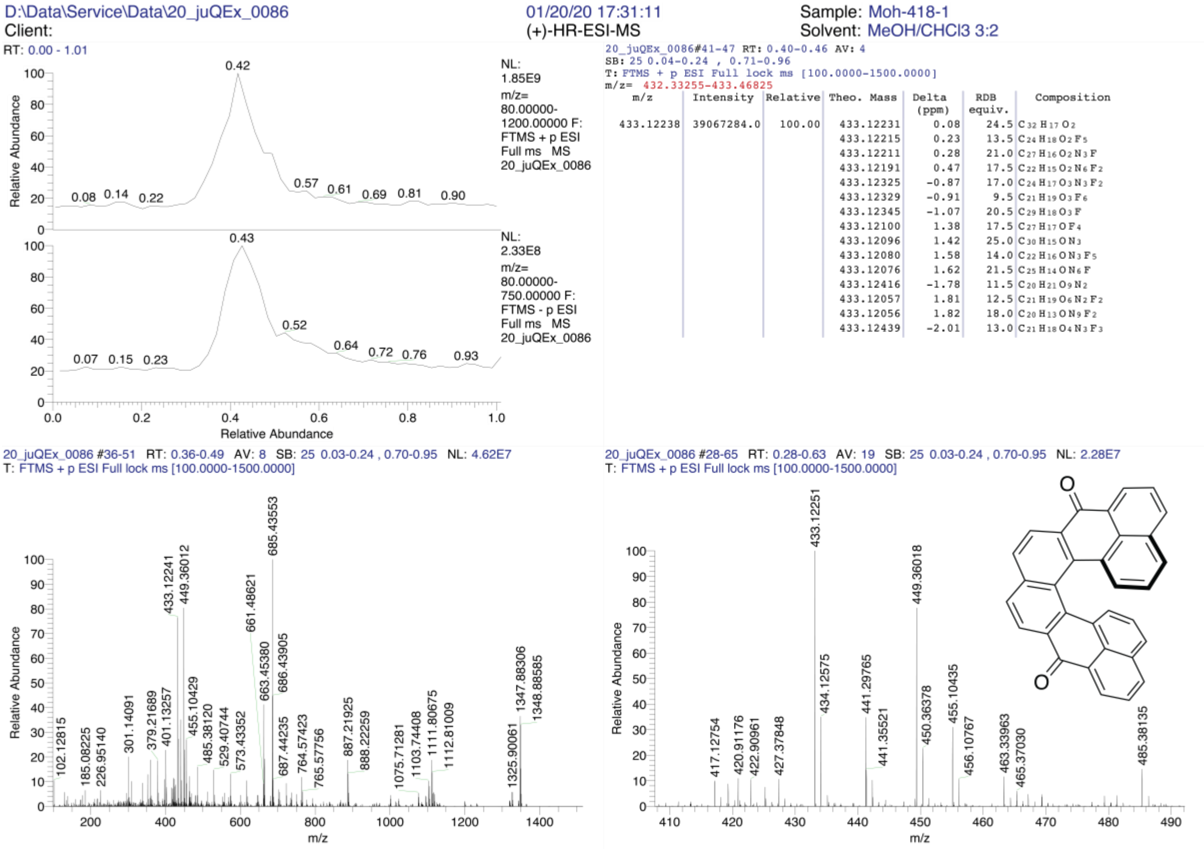

m/z	Intensity	Relative	Theo. Mass	Delta (ppm)	RDB equiv.	Composition
433.12238	39067284.0	100.00	433.12231	0.08	24.5	$C_{32}H_{17}O_2$
			433.12215	0.23	13.5	$C_{24}H_{18}O_2F_5$
			433.12211	0.28	21.0	$C_{27}H_{16}O_2N_3F$
			433.12191	0.47	17.5	$C_{22}H_{15}O_2N_6F_2$
			433.12325	-0.87	17.0	$C_{24}H_{17}O_3N_3F_2$
			433.12329	-0.91	9.5	$C_{21}H_{19}O_3F_6$
			433.12345	-1.07	20.5	$C_{29}H_{18}O_3F$
			433.12100	1.38	17.5	$C_{27}H_{17}OF_4$
			433.12096	1.42	25.0	$C_{30}H_{15}ON_3$
			433.12080	1.58	14.0	$C_{22}H_{16}ON_3F_5$
			433.12076	1.62	21.5	$C_{25}H_{14}ON_6F$
			433.12416	-1.78	11.5	$C_{20}H_{21}O_9N_2$
			433.12057	1.81	12.5	$C_{21}H_{19}O_6N_2F_2$
			433.12056	1.82	18.0	$C_{20}H_{13}ON_9F_2$
			433.12439	-2.01	13.0	$C_{21}H_{18}O_4N_3F_3$

Figure S17. HRMS (ESI): benzo[*no*]phenaleno[1,2-*a*]tetraphene-4,9-dione (**8**).

4 X-ray crystallography

Table S1. Crystal data and structure refinement for **7** (CCDC no. 2289143).

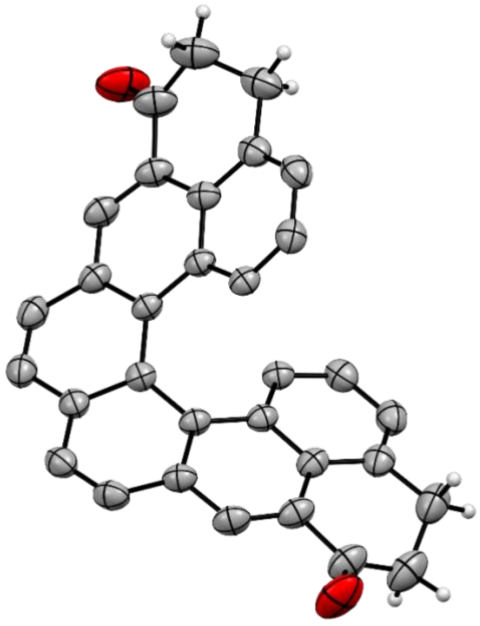

Empirical formula	$C_{33}H_{22}Cl_2O_2$
Formula weight	521.40
Temperature / K	160(1)
Crystal system	monoclinic
Space group	I2/a
a / Å	12.7528(3)
b / Å	8.7941(3)
c / Å	21.9849(7)
α / °	90
β / °	98.937(3)
γ / °	90
Volume / Å^3	2435.66(13)
Z	4
ρ_{calc} / g cm^{-3}	1.422
μ / mm^{-1}	2.639
F(000)	1080.0
Crystal size / mm^3	0.26 × 0.07 × 0.02
Radiation	CuKα (λ = 1.54184)
2θ range for data collection / °	8.142 to 148.944
Index ranges	$-15 \leq h \leq 15$, $-10 \leq k \leq 9$, $-27 \leq l \leq 27$
Reflections collected	12751
Independent reflections	2485 [R_{int} = 0.0338, R_{sigma} = 0.0309]
Data/restraints/parameters	2485 / 52 / 210
Goodness-of-fit on F^2	1.073
Final *R* indexes [$I \geq 2\sigma(I)$]	R_1 = 0.0706, wR_2 = 0.2148
Final *R* indexes [all data]	R_1 = 0.0786, wR_2 = 0.2242
Largest diff. peak/hole / e Å^{-3}	0.61 / –0.52

Table S2. Crystal data and structure refinement for 2*H*-**1** (CCDC no. 2289144).

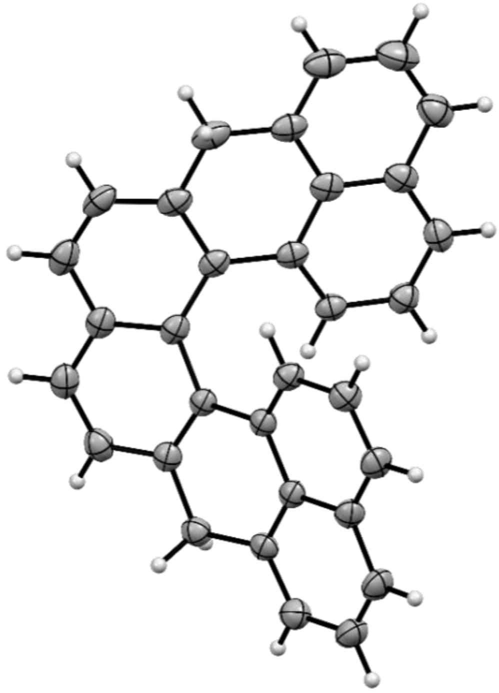

Empirical formula	$C_{32}H_{20}$
Formula weight	404.48
Temperature / K	160(1)
Crystal system	triclinic
Space group	P-1
a / Å	12.4425(8)
b / Å	13.5187(10)
c / Å	13.7179(6)
α / °	90.599(5)
β / °	106.438(5)
γ / °	113.639(7)
Volume / Å^3	2007.2(2)
Z	4
ρ_{calc} / g cm^{-3}	1.339
μ / mm^{-1}	0.576
F(000)	848.0
Crystal size / mm^3	0.16 × 0.04 × 0.02
Radiation	CuKα (λ = 1.54184)
2θ range for data collection / °	6.786 to 136.494
Index ranges	$-14 \le h \le 14$, $-16 \le k \le 16$, $-16 \le l \le 14$
Reflections collected	27569
Independent reflections	7339 [R_{int} = 0.0717, R_{sigma} = 0.0628]
Data/restraints/parameters	7339 / 0 / 577
Goodness-of-fit on F^2	0.999
Final *R* indexes [$I \ge 2\sigma(I)$]	R_1 = 0.0528, wR_2 = 0.1315
Final *R* indexes [all data]	R_1 = 0.0946, wR_2 = 0.1572
Largest diff. peak/hole / e Å^{-3}	0.23 / –0.23

Table S3. Crystal data and structure refinement for **8** (CCDC no. 2446997).

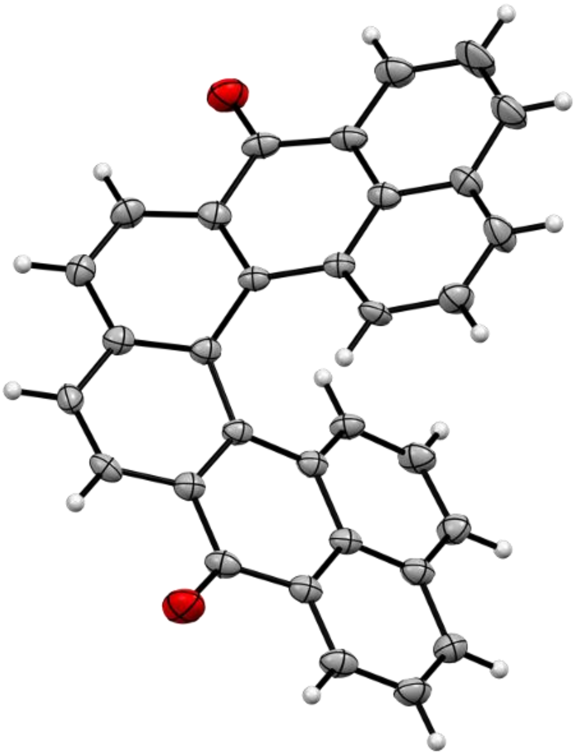

Empirical formula	$C_{32}H_{16}O_2$
Formula weight	432.45
Temperature / K	160(1)
Crystal system	monoclinic
Space group	$P2_1$
a / Å	10.2463(3)
b / Å	14.4368(4)
c / Å	13.5947(3)
α / °	90
β / °	91.638(3)
γ / °	90
Volume / Å^3	2010.16(10)
Z	4
ρ_{calc} / g cm^{-3}	1.429
μ / mm^{-1}	0.696
F(000)	896.0
Crystal size / mm^3	0.09 × 0.06 × 0.03
Radiation	CuKα (λ = 1.54184)
2θ range for data collection / °	6.504 to 148.938
Index ranges	$-12 \le h \le 12$, $-18 \le k \le 18$, $-16 \le l \le 16$
Reflections collected	31676
Independent reflections	8185 [R_{int} = 0.0417, R_{sigma} = 0.0339]
Data/restraints/parameters	8185/1/613
Goodness-of-fit on F^2	1.028
Final R indexes [$I \ge 2\sigma(I)$]	R_1 = 0.0766, $wR2$ = 0.1825
Final R indexes [all data]	R_1 = 0.0950, $wR2$ = 0.2045
Largest diff. peak/hole / e Å^{-3}	0.85/–0.25

checkCIF/PLATON report for CCDC no. 2446997 (compound 8)

Alert level B: Large Reported Max. (Positive) Residual Density 0.85 e A^{-3}

Answer: After the final refinements of the crystal structure of compound **8**, the highest residual electron density peak is observed at 0.85 electrons (causing a B-alert in the checkCIF report). It is not an isolated peak, for instance, the ten highest residual electron density peaks are above 0.6 electrons. The intensity and location of those peaks suggest a small disorder of the molecules in the crystal, but it is not considered significant enough to be taken into account in the refined model.

5 Additional STM data

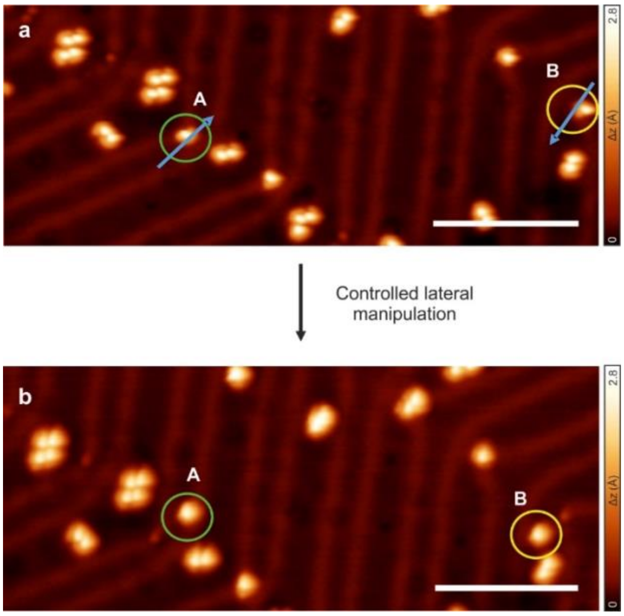

Figure S18. Lateral manipulation process for two molecules **1**, namely A and B, located at the elbow position of the herringbone reconstruction of the Au(111) surface, as marked with the colored circles. Controlled tip-induced lateral manipulation can be used to move the molecule out of the reactive elbow site without altering the molecular structure. Scanning parameters: $V_b = -0.2$ V, $I_t = 20$ pA by adjusting $I_t \sim 0.35$ nA with Z-offset. Scanning parameters in STM images (**a**, **b**): $V_b = -0.2$ V, $I_t = 20$ pA, scale bars = 10 nm. The arrows indicate the displacement of the molecules by the lateral manipulation process.

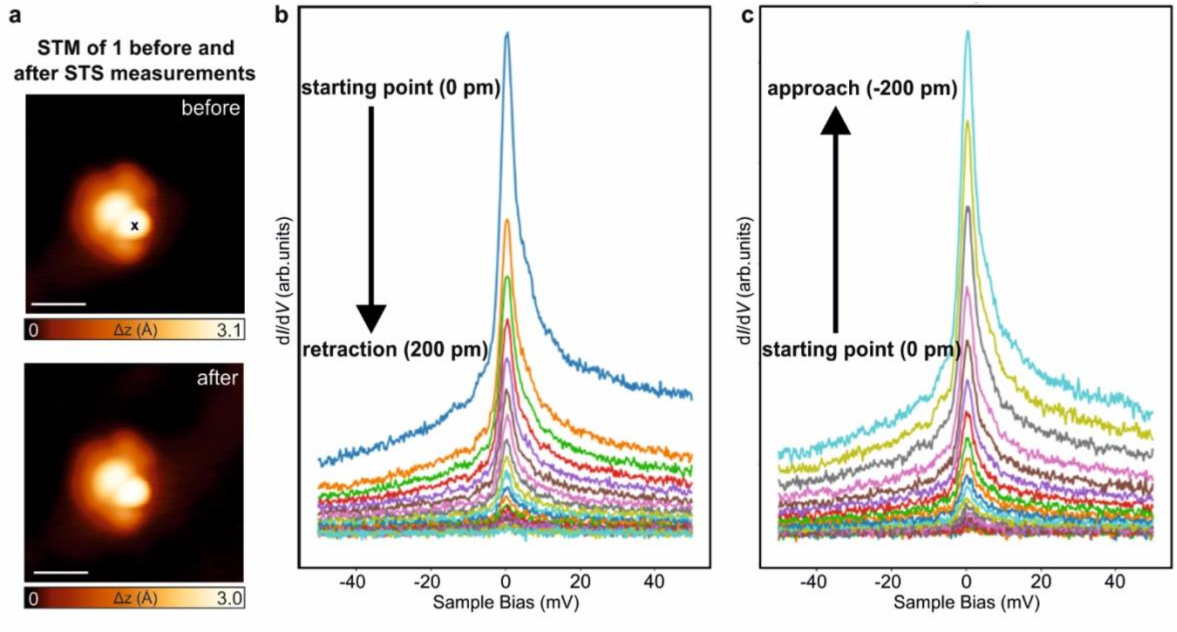

Figure S19. Vertical approach/retraction to **1** while acquiring STS measurements. **a** Constant-current STM images of **1** as obtained after the on-surface generation of the diradical before and after both approach/retraction events. Scanning parameters: V_b = −0.2 V, I_t = 20 pA, scale bars = 1 nm. **b** Vertical retraction of the tip while recording the spin state of **1**. The STM tip is stabilized on the molecule in the position marked by the black cross in **a** at a starting height indicated as 0 pm determined by the setpoint parameters. The dI/dV spectra were acquired while retracting the tip in steps of 10 pm from 0 to 200 pm. **c** Vertical approach of the tip while recording the spin state of **1** as described in **b**. All the STM images and STS measurements were acquired with a CO-functionalized tip. Spectroscopy parameters: V_b = 50 mV, I_t = 500 pA and a root mean squared modulation voltage V_{rms} = 0.8 mV.

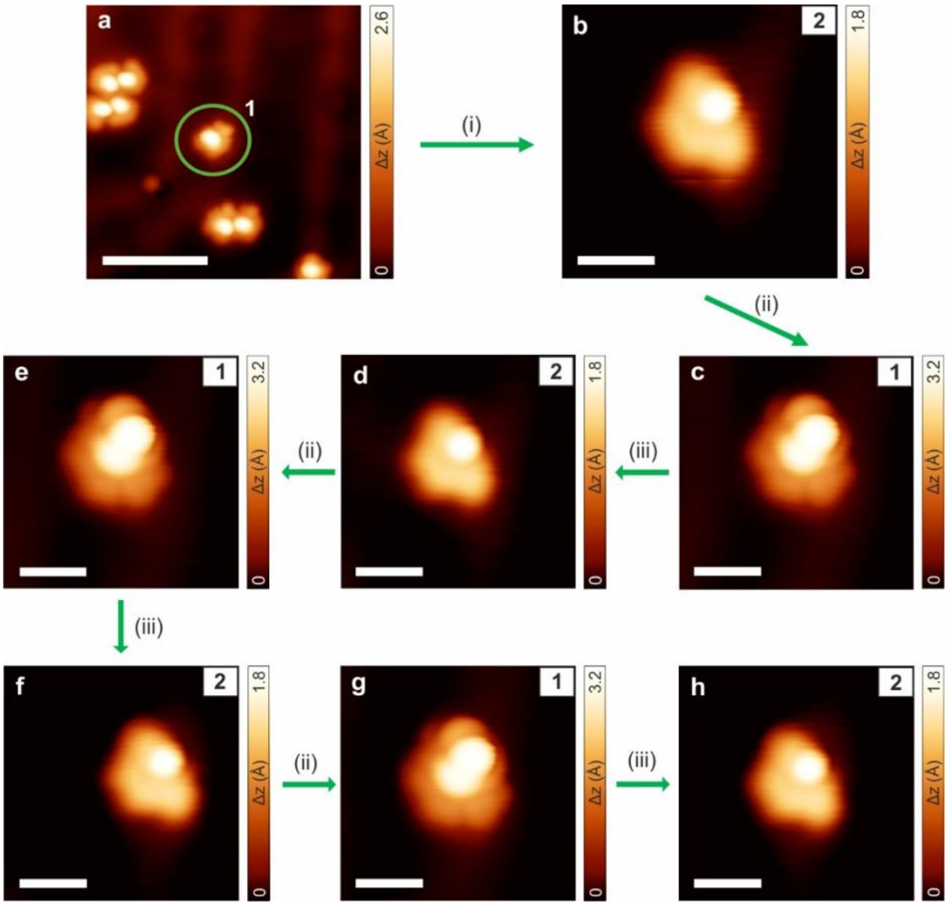

Figure S20. Reversible multiple magnetic switching from **1** to **2**. **a** Molecule **1** out of the elbow site, in an fcc region (highlighted by the green circle). Scanning parameters: $V_b = -0.2$ V, $I_t = 20$ pA, scale bar = 4 nm. **b** Specie **2** obtained from the molecule **1** in panel **a**. Scanning parameters: $V_b = -0.2$ V, $I_t = 20$ pA. **a–h** Specific scanning conditions enable the reversible transformation from **1** to **2**, with the following parameters: i) $V_b = -3.0$ V, $I_t = 20$ pA, ii) $V_b = -1.5$ V, $I_t = 20$ pA, iii) $V_b = 0.005$ V, $I_t = 20$ pA. Scanning parameters: $V_b = -1.5$ V, $I_t = 20$ pA (**c**, **e**, **g**), $V_b = -0.2$ V, $I_t = 20$ pA for (**d**, **f**, **h**). Scale bars = 1 nm (**b–h**).

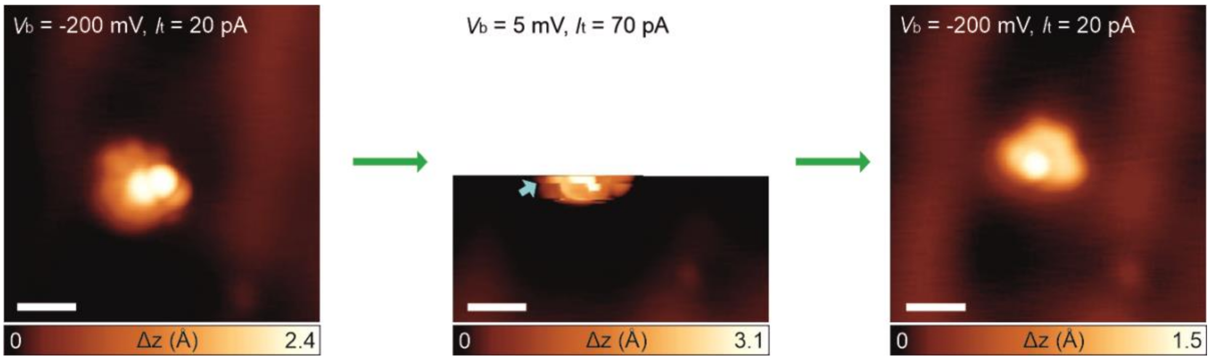

Figure S21. Displacement of **1** before being transformed into **2** under certain scanning conditions. Left: STM image before transformation; middle: STM image showing the precise moment when the molecule is displaced during the scan; right: STM image after the transformation into **2** has taken place. The blue arrow depicted in the middle panel highlights the displacement of the molecules. All scale bars = 1 nm.

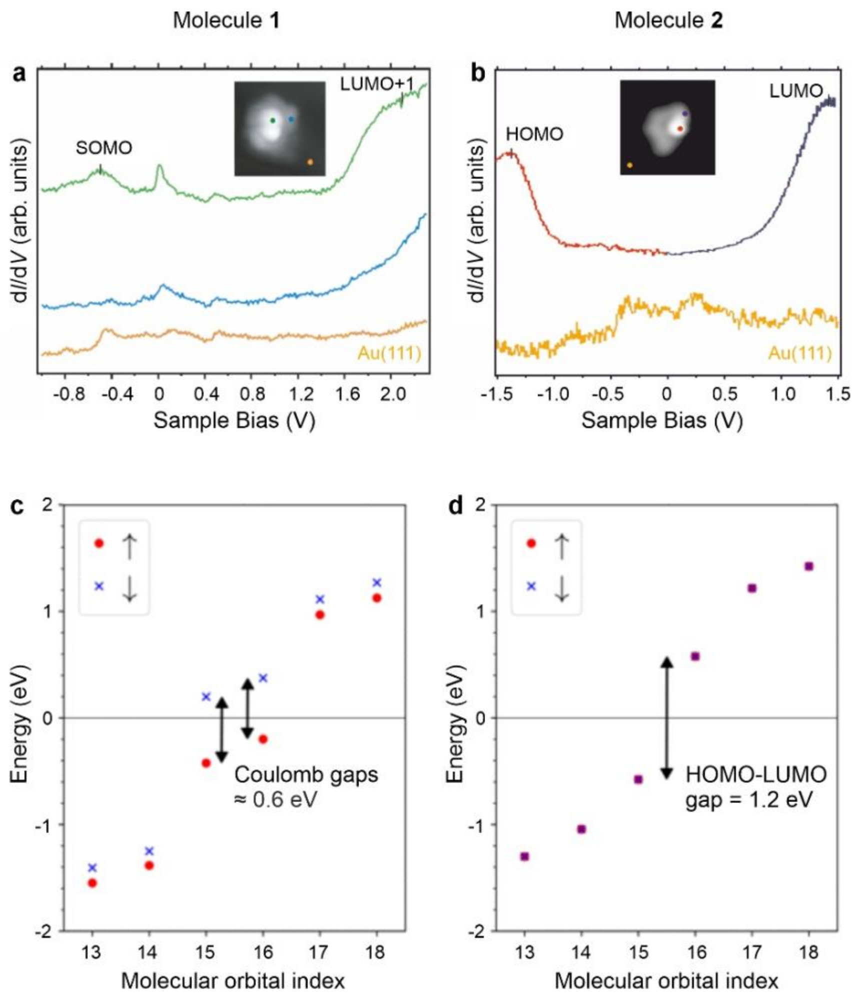

Figure S22. Long-range scanning tunneling spectroscopy of species **1** and **2**. **a** Differential conductance spectra on selected positions of **1**; the positions at which the spectra on the molecule were acquired are highlighted in the inset STM topography image with filled blue and green circles. Open feedback parameters: V_b= 2.3 V, I_t = 100 pA, V_{rms} = 20 mV. **b** Differential conductance spectra on selected positions of **2**. The purple and red circles highlight the positions at which the spectra were acquired. Open feedback parameters: (positive region) V_b= –1.0 V, I_t = 100 pA, V_{rms} = 20 mV; (negative region) V_b= 1.0 V, I_t =100 pA, V_{rms} = 20 mV. The orange circles in the inset images of **a** and **b** correspond to the reference dI/dV spectra acquired on Au(111). **c,d** Energy levels of molecule **1** (**c**) and molecule **2** (**d**), obtained for the ground-state solution of the corresponding MFH models (see Methods for details). Solid black line denotes the Fermi level.

For species **2**, the gap corresponds to the conventional energy difference between the highest occupied molecular orbital (HOMO) and the lowest unoccupied molecular orbital (LUMO) (HOMO–LUMO gap) in closed-shell molecules, determined by the hybridisation of the two zero modes (see panel **d**). In contrast, species **1** is an open-shell system with two ferromagnetically coupled spins in two singly occupied molecular orbitals (SOMOs). In this case, the gap is not governed by hybridisation but instead by the Coulomb gap, which must be overcome to add an additional electron to a SOMO (see panel **c**). The experimental scanning tunneling spectroscopy (STS) results detect the HOMO and LUMO states for species **2** at –1.3 V and +1.4 V, respectively. In the case of molecule **1**, instead, we revealed the SOMO at –0.5 V and the LUMO+1 at +2.1 V, while the SUMO was not experimentally detected. Both the experimental and theoretical results confirm that the Coulomb gap is smaller than the HOMO–LUMO gap.

6 Additional computational data

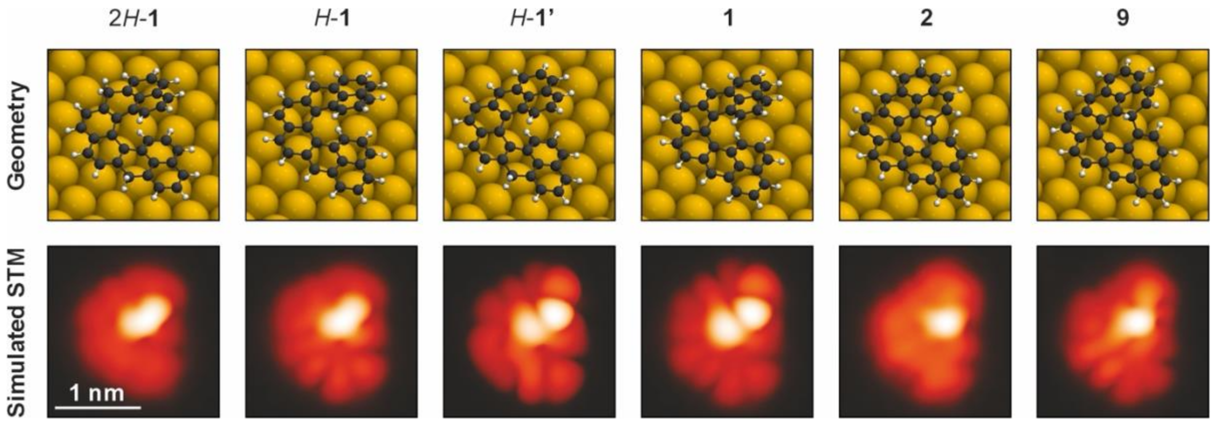

Figure S23. DFT-optimized geometries of the main species discussed in this work, along with their simulated STM images reported in Fig. 2, bottom in the main text.

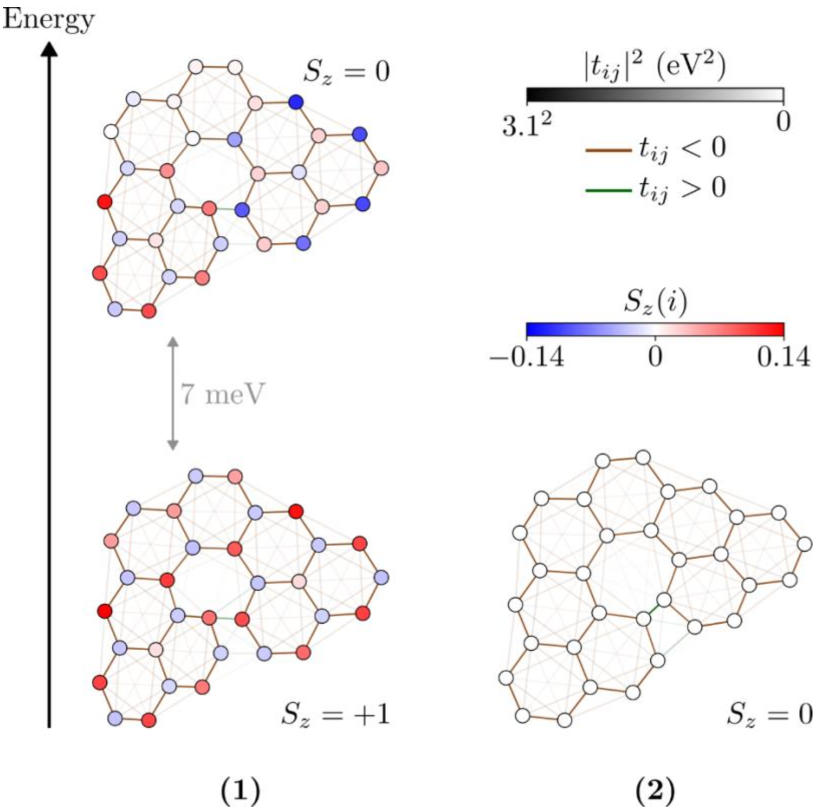

Figure S24. MFH calculations (see Methods for details) for molecules **1** and **2**. Molecule **1** features a triplet ground state followed by a singlet antiferromagnetic state 7 meV higher in energy, suggesting a diradical character with ferromagnetic interactions. Molecule **2** features a singlet non-magnetic ground state, suggesting a closed-shell character.

7 References

[1] Ravat, P. *et al.* Cethrene: A helically chiral biradicaloid isomer of heptazethrene. *Angew. Chem. Int. Ed.* **55**, 1183–1186 (2016).

[2] Park, S. H., Kim, J. Y. & Chang, S. Rhodium-catalyzed selective olefination of arene esters via C−H bond activation. *Org. Lett.* **13**, 2372–2375 (2011).

[3] Církva, V. *et al.* Preparation and physicochemical properties of [6]helicenes fluorinated at terminal rings. *J. Org. Chem.* **84**, 1980–1993 (2019).

[4] Fulmer, G. R. *et al.* NMR chemical shifts of trace impurities: Common laboratory solvents, organics, and gases in deuterated solvents relevant to the organometallic chemist. *Organometallics* **29**, 2176–2179 (2010).
